# Supplementary material for: Pangenome and pantranscriptome as the new reference for gene-family characterization: A case study of basic helix-loop-helix (bHLH) genes in barley
Source: Plant Commun. 2024 Nov 9;6(1):101190. doi: 10.1016/j.xplc.2024.101190 (PMC11783906; doi:10.1016/j.xplc.2024.101190)

Plant Communications, Volume *6*

Supplemental information

Pangenome and pantranscriptome as the new reference for gene-family characterization: A case study of basic helix-loop-helix (*bHLH*) genes in barley

Cen Tong, Yong Jia, Haifei Hu, Zhanghui Zeng, Brett Chapman, and Chengdao Li

Supplementary Figures

Supplementary Figure S1. Displays the protein motifs and gene structures of *bHLHs* in barley. **A**. Displays the top 10 conserved protein motifs. Scale bar indicates amino acid (aa) sequence length. **B**. Displays the gene structure (exon and intron). Scale bar indicates gene sequence length in thousand base pair (kb). Target *bHLHs* were sorted by a maximum likelihood phylogenetic tree (left), with different subfamilies highlighted in same colors as in Figure 2.

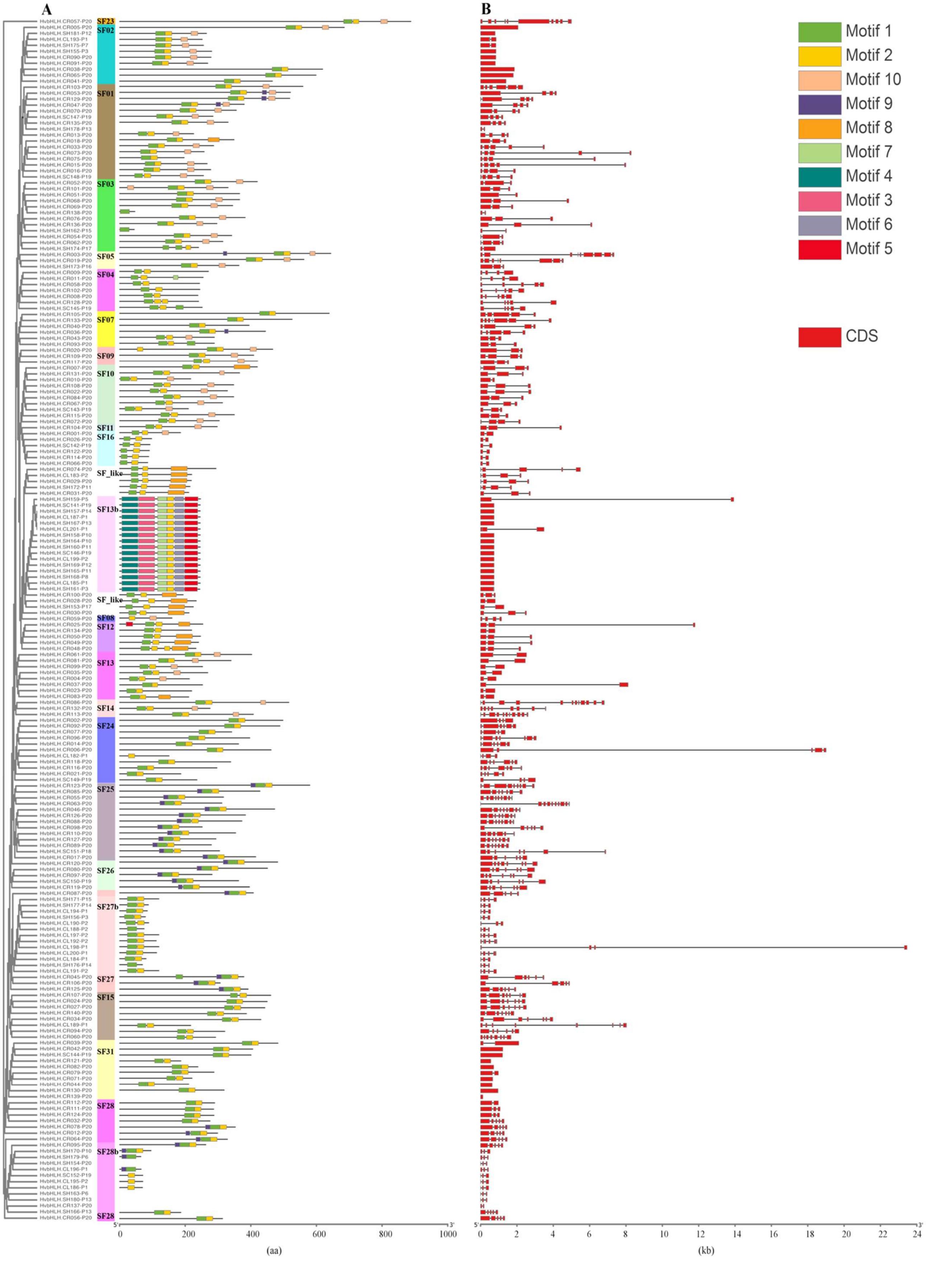

Supplementary Figure S2. Displays the chromosome location, synteny relationship, and gene duplication type of bHLHs in Morex. bHLHs located in conserved collinear regions were linked by red line. Based on gene duplication mechanisms, bHLHs were classified as whole-genome/segmental duplication (purple), tandem duplication (green), proximal duplication (pink), and dispersed duplication (blue). Newly predicted bHLH gene IDs in this study were highlighted in red.

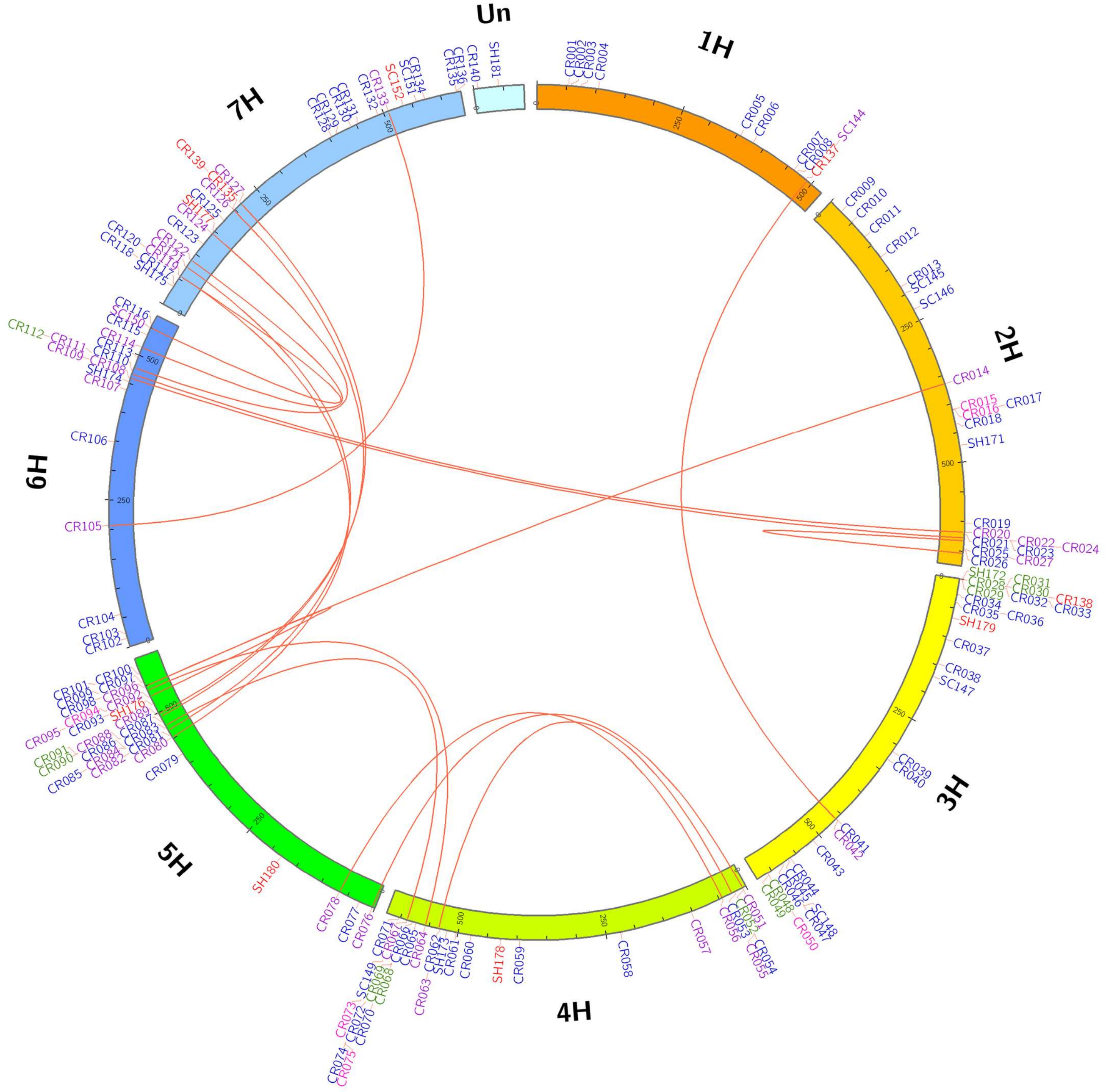

Supplementary Figure S3. Displays the distribution of *Ka*, *Ks*, and *Ka*/*Ks* for each *bHLH* OG (sorted by evolutionary phylogeny). See supplementary file S13 for detailed calculation.

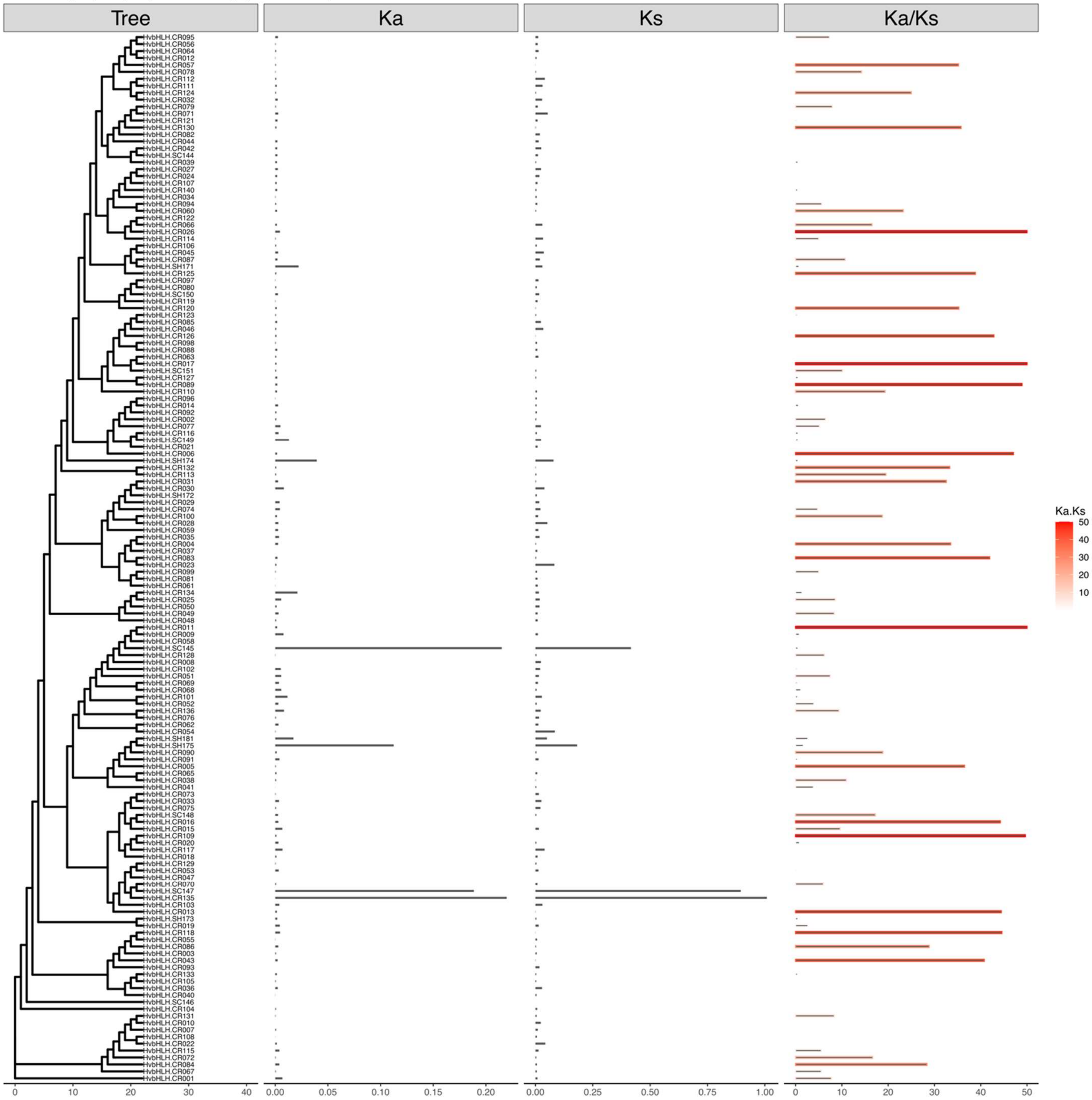

Supplement: Document S1. Supplemental Figures 1–3 [file mmc1.pdf]
