# Supplementary material for: Pangenome and pantranscriptome as the new reference for gene-family characterization: A case study of basic helix-loop-helix (bHLH) genes in barley
Source: Plant Commun. 2024 Nov 9;6(1):101190. doi: 10.1016/j.xplc.2024.101190 (PMC11783906; doi:10.1016/j.xplc.2024.101190)

Plant Communications
Resource article

CellPress
Partner Journal

Pangenome and pantranscriptome as the new reference for gene-family characterization: A case study of basic helix-loop-helix (*bHLH*) genes in barley

Cen Tong[1,2,6], Yong Jia[1,2,6,*], Haifei Hu[1,2], Zhanghui Zeng[3], Brett Chapman[1,2] and Chengdao Li[1,2,4,5,*]

[1]Western Crop Genetic Alliance, Murdoch University, Murdoch, WA 6150, Australia

[2]State Agricultural Biotechnology Centre (SABC), College of Science, Health, Engineering and Education, Murdoch University, Murdoch, WA 6150, Australia

[3]College of Life and Environmental Sciences, Hangzhou Normal University, Hangzhou 311121, China

[4]Department of Primary Industry and Regional Development, Government of Western Australia, South Perth, WA 6155, Australia

[5]College of Agriculture, Shandong Agricultural University, TaiAn, China

[6]These authors contributed equally to the article.

***Correspondence: Yong Jia (y.jia@murdoch.edu.au), Chengdao Li (c.li@murdoch.edu.au)**

https://doi.org/10.1016/j.xplc.2024.101190

ABSTRACT

Genome-wide identification and comparative gene-family analyses have commonly been performed to investigate species-specific evolution linked to various traits and molecular pathways. However, most previous studies have been limited to gene screening in a single reference genome, failing to account for the gene presence/absence variations (gPAVs) in a species. Here, we propose an innovative pangenome-based approach for gene-family analyses based on orthologous gene groups (OGGs). Using the basic helix-loop-helix (bHLH) transcription factor family in barley as an example, we identified 161–176 *bHLHs* in 20 barley genomes, which can be classified into 201 OGGs. These 201 OGGs were further classified into 140 core, 12 softcore, 29 shell, and 20 line-specific/cloud *bHLHs*, revealing the complete profile of *bHLH* genes in barley. Using a genome-scanning approach, we overcame the genome annotation bias and identified an average of 1.5 un-annotated core *bHLHs* per barley genome. We found that whole-genome/segmental duplicates are predominant mechanisms contributing to the expansion of most core/softcore *bHLHs*, whereas dispensable *bHLHs* are more likely to result from small-scale duplication events. Interestingly, we noticed that the dispensable *bHLHs* tend to be enriched in the specific subfamilies SF13, SF27, and SF28, implying the potentially biased expansion of specific *bHLHs* in barley. We found that 50% of the *bHLHs* contain at least 1 intact transposon element (TE) within the 2-kb upstream-to-downstream region. *bHLHs* with copy-number variations (CNVs) have 1.48 TEs on average, significantly more than core *bHLHs* without CNVs (1.36), supporting a potential role of TEs in *bHLH* expansion. Analyses of selection pressure showed that dispensable *bHLHs* have experienced clear relaxation of selection compared with core *bHLHs*, consistent with their conservation patterns. We also integrated the pangenome data with recently available barley pantranscriptome data from 5 tissues and discovered apparent transcriptional divergence within and across *bHLH* subfamilies. We conclude that pangenome-based gene-family analyses can better describe the previously untapped, genuine evolutionary status of *bHLHs* and provide novel insights into *bHLH* evolution in barley. We expect that this study will inspire similar analyses in many other gene families and species.

Key words: basic helix-loop-helix, bHLH, barley pangenome, core and dispensable genes, genome-wide gene-family evolution, orthologous gene group, pantranscriptome

Tong C., Jia Y., Hu H., Zeng Z., Chapman B., and Li C. (2025). Pangenome and pantranscriptome as the new reference for gene-family characterization: A case study of basic helix-loop-helix (*bHLH*) genes in barley. Plant Comm. **6**, 101190.

Published by the Plant Communications Shanghai Editorial Office in association with Cell Press, an imprint of Elsevier Inc., on behalf of CSPB and CEMPS, CAS.

© 2024 The Authors.
This is an open access article under the CC BY license (http://creativecommons.org/licenses/by/4.0/).

INTRODUCTION

Driven by the ever-decreasing cost of genome sequencing, the number of species with sequenced reference genomes has increased exponentially in the last 2 decades (Marks et al., 2021). The availability of complete genomic sequences for more and more species has made genome-wide characterization of various gene families a routine analysis for researchers. This has led to the publication of hundreds or even thousands of genome-wide gene-family characterization studies. Take, for example, the basic helix-loop-helix (*bHLH*) gene family, one of the most highly expanded transcription factor (TF) families (Hao et al., 2021): a preliminary literature search on May 30, 2024 revealed more than 65 such studies in plants (Supplemental Data 1), spanning 53 species from diverse orders and families. One major aim of these genome-wide studies is to investigate the species-specific patterns of gene evolution, which may underlie plants' genetic and phenotypic diversity. In crop species, the related phenotypes may correspond to important agronomic traits such as grain yield, nutrient content, and processing quality, depending on the particular biological function of the target gene (Haas et al., 2019). For evolutionarily important species, these gene-family analyses may provide critical insights into the evolution of plant genomes and phenotypes (Carretero-Paulet et al., 2010; Zhou et al., 2020).

Among plant TFs, the bHLHs rank second after the MYBs in family size, representing one of the most highly expanded TF families in the plant genome (Feller et al., 2011). The bHLH domain (∼60 amino acids [aa] in length) comprises 2 conserved aa motifs, a basic aa region and an HLH region, but the region outside of the bHLH domain can vary greatly (Toledo-Ortiz et al., 2003). Within the bHLH domain, the N-terminal basic region and the C-terminal HLH region are responsible for DNA binding and protein dimerization, respectively (Toledo-Ortiz et al., 2003). The basic DNA-binding regions of different bHLH subfamilies may recognize different nucleic acid sites in gene promoter regions and regulate target-gene expression. According to previous functional characterization studies, bHLH-encoding genes play critical roles in various plant growth and developmental processes, such as seed germination, flowering-time control, and cell-fate determination (Hao et al., 2021). In addition, *bHLH*s have also been shown to play an important role in adaptive responses to various stress factors, including nutrient deficiency and high salinity, as well as in light signaling and phytohormone responses (Sun et al., 2018; Hao et al., 2021). In particular, the functions of *bHLH*s in plant environmental adaptation have attracted increasing attention from plant biologists and crop breeders (Wang et al., 2018a; Li et al., 2020; Miao et al., 2020; Wang et al., 2020; Hao et al., 2021).

In plant genomes in which the *bHLH* gene family has been characterized, the number of identified *bHLH*s varies significantly. For example, 162 bHLHs have been reported in *Arabidopsis* (Bailey et al., 2003), 167 in rice (Li et al., 2006), 208 in maize (Zhang et al., 2018), 141 in barley (Ke et al., 2020), 159 in tomato (Sun et al., 2015), 124 in potato (Wang et al., 2018b), 602 in canola (Ke et al., 2020), 142 in cucumber (Li et al., 2020), 122 in pepper (Zhang et al., 2020), and 85 in ginkgo (Zhou et al., 2020). Up to 32 *bHLH subfamilies have been identified on the basis of* phylogenetic analyses in these studies (Carretero-Paulet et al., 2010). Comparative *bHLH* gene-family analyses have frequently enabled the identification of species-specific evolutionary patterns (Carretero-Paulet et al., 2010; Miao et al., 2020; Ndayambaza et al., 2021), which seems to be a common observation for *bHLH*s.

Variations in gene-family size and subfamily conservation across species contain critical information that may help us understand plant evolutionary history and environmental adaptation (Hanada et al., 2008; Robertson et al., 2017). Although copy-number variations (CNVs) are commonly identified and characterized in pangenome studies, most systematic genome-wide identification and gene-family evolution studies are still based on the gene content of a single reference genome. For the bHLHs, no pangenome-wide gene-family analyses have been performed in any plant species to date (Supplemental Data 1). Because a reference genome only covers the gene content of a single individual, it cannot reflect the genetic diversity of a population, particularly gene presence/absence variations (gPAVs) and CNVs. The term pangenome, which has been widely adopted in genome studies of different organisms, reflects the observation that genes often display gPAVs across different lines in the same species (Tettelin et al., 2005). Recent pangenome studies in plants have revealed that gPAVs and CNVs are common in the genomes of various species (Swanson-Wagner et al., 2010; Bayer et al., 2020; Jayakodi et al., 2020). On the basis of their conservation levels, genes in a pangenome can be categorized as core (strictly conserved) or dispensable/variable (absent in at least 1 genome) (Gao et al., 2019; Bayer et al., 2020; Sun, 2022), and these classifications are suggested to have important biological implications. However, most gene-family studies have been performed on the gene content of a reference genome only, and little attention has been paid to pangenome-wide analyses of specific gene families.

Taking advantage of published data from 20 barley pangenome accessions (Jayakodi et al., 2020), the present study aims to overcome the limitation of a single reference genome and to characterize the complete evolutionary status of a gene family in a target species. We propose an innovative pangenome-based approach, which involves scanning genes in all pangenome assemblies and identifying orthologous gene groups (OGGs) for gene-family characterization. We chose the bHLH TFs, one of the most highly expanded TF families in plants, to illustrate the unique features of pangenome-based gene-family analyses. Pangenome OGGs were used for phylogenetic, gene-structure, PAV, CNV, natural selection, and transcriptome analyses. To eliminate gene-number variations caused by differences in genome annotation quality, we used an algorithm implemented by BITACORA (Vizueta et al., 2020), which scans a genomic sequence and enables the identification of target genes not covered by the genome annotation. We also introduced the idea of core and dispensable genes to gene-family analyses, providing novel insights into gene-family evolution. We demonstrate that pangenome-based analyses of gene families have clear advantages over traditional studies based on reference genomes for describing the genuine evolutionary status of a gene family in a target species.

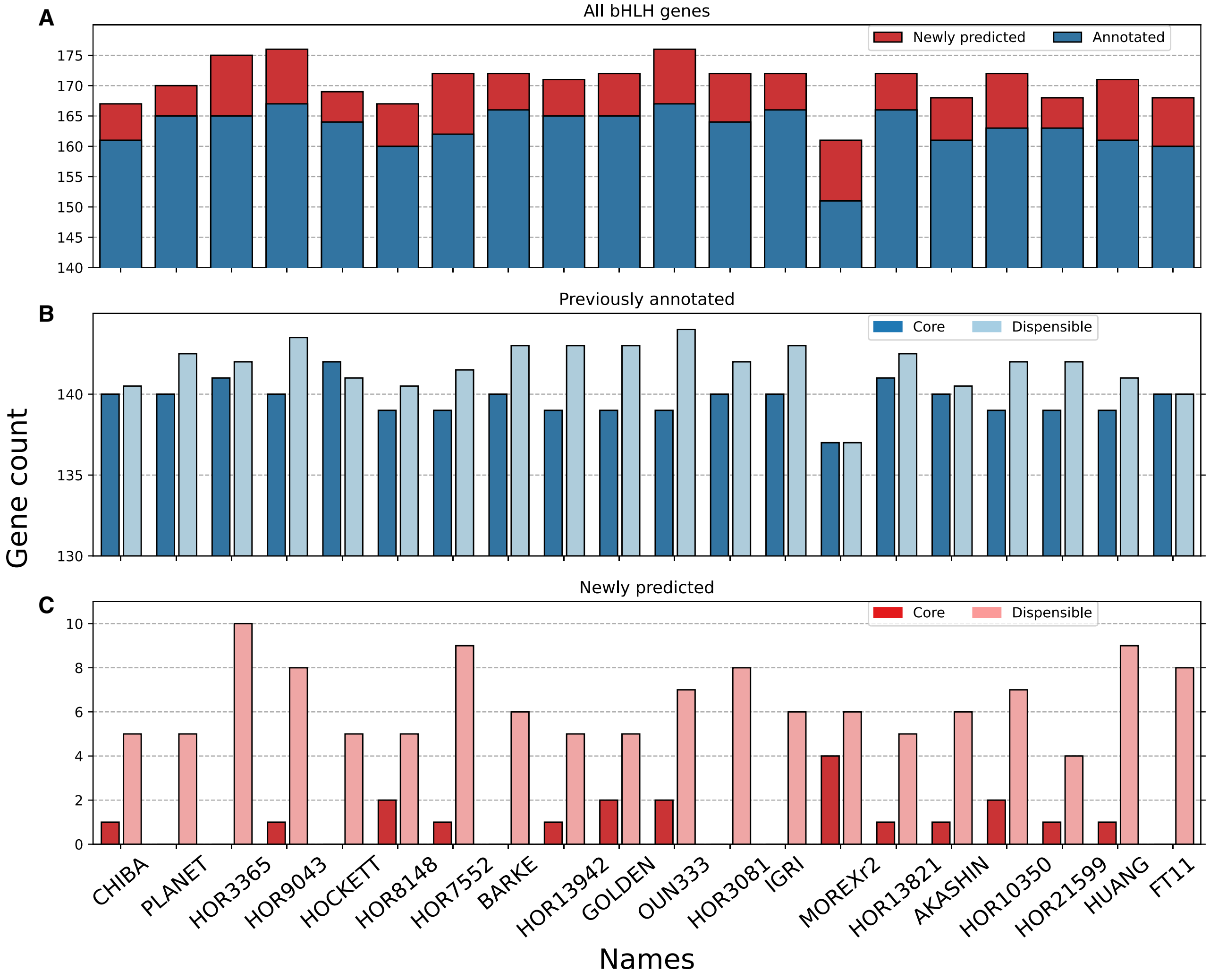

Figure 1. Identification of *bHLHs* in the barley pangenome

(A) Distribution of annotated and newly predicted *bHLHs* in 20 barley genome assemblies.

(B) Distribution of core and dispensable *bHLH*s in annotated genes.

(C) Distribution of core and dispensable *bHLH*s in newly predicted genes. Only those *bHLH*s with a bHLH domain longer than 36 aa were counted. See Supplemental Data 4 for detailed sequences and ID information.

RESULTS

Identification of *bHLHs* in 20 barley pangenome assemblies

To obtain the complete profile of bHLH-encoding genes in the barley pangenome, we used an algorithm implemented by BITACORA (Vizueta et al., 2020), which scans the whole-genome assembly using both reference bHLH protein sequences and the bHLH domain (Pfam: PF00010; E-value threshold $\leq$1e−5) and enables the identification of novel, unannotated *bHLH* gene sequences. The identified bHLH hits were further filtered for the presence of a complete bHLH domain. To determine the domain length threshold, we retrieved previously well-curated rice bHLH homologs from the Rice Genome Annotation Project (RGAP) database and extracted the full bHLH domain sequences on the basis of HMMER scan, which were found to range from 36 aa to 82 aa (Supplemental Data 2). Because of potential species variation in bHLHs, we relaxed the bHLH domain threshold to $\geq$30 aa to avoid missing any potential targets. After filtration, the total number of *bHLH*s identified in the 20 barley genomes ranged from 161 (Morex version 2) to 176 (HOR3365 and OUN333) (Figure 1A; Supplemental Data 3 and 4), revealing clear variations in *bHLH* gene number across the barley pangenome. Although we used a domain length threshold $\geq$30 aa, validation of bHLH hits showed that the minimum domain length of these hits was 36 aa, consistent with that of the curated rice bHLHs. Among the identified bHLH genes, 151 (Morex version 2) to 167 (HOR3365 and OUN333) *bHLH*s corresponded to previously annotated genes (Figure 1A), whereas the number of newly predicted *bHLH*s ranged from 5 to 10 across the 20 barley genomes (Figure 1A).

The full aa sequence length for the identified bHLHs ranged from 36 to 888 aa (Supplemental Data 4), compared with 68 to 930 aa for the curated rice bHLHs (Supplemental Data 2). We noted that several newly predicted bHLH sequences were relatively short and may represent partial sequences that cover only the bHLH

domain. The identification of such partial bHLHs is expected, as only the bHLH domain of bHLH TFs is typically conserved, and sequences outside of this domain exhibit extremely low (if any) conservation (Carretero-Paulet et al., 2010). These newly predicted short bHLHs lack sequences outside the bHLH domain, which would not be identified if they were not present in the reference *bHLH*s. We observed direct support for this hypothesis when comparing the prediction results between Morex version 3 and Morex version 2. A total of 161 *bHLH*s (151 annotated and 10 newly predicted) were identified in Morex version 2, compared with 161 *bHLH*s (154 annotated and 7 newly predicted) in Morex version 3 (Supplemental Data 5). Although most *bHLHs* were identical between Morex versions 2 and 3, 4 pairs of *bHLH*s (cluster 131, cluster 141, cluster 147, and cluster 152 in Supplemental Data 5) were annotated as full length in one version but were newly predicted as partial sequences in the other version. The "mutual verification" of these newly predicted *bHLH*s between Morex versions 2 and 3 annotations further highlighted the advantage and accuracy of the genome-scanning approach implemented in our study, which could overcome the genome annotation bias and produce consistent, reliable, and exhaustive gene searches based on the genomic sequence. The prediction results from Morex version 2 were used for downstream analyses.

Core and dispensable *bHLHs*

To investigate the conservation of *bHLH*s in the barley pangenome, the *bHLH*s identified in 20 barley varieties were assigned to different OGGs. Using an aa similarity threshold of $\geq$95%, the 3411 *bHLH*s from the 20 barley genomes were divided into 201 OGGs (Supplemental Data 6). The number of OGGs was much higher than the number of *bHLH*s in the Morex reference genome (161) and the average number of *bHLH*s per genome (170), suggesting the presence of PAVs for *bHLH*s in the barley pangenome. On the basis of the PAV of each OGG in the 20 barley genomes, the 201 *bHLH* OGGs were classified into 140 core *bHLH*s (present in all 20 varieties) and 61 dispensable *bHLH*s (absent in at least one variety), including 12 softcore (conserved in $\geq$90% lines), 29 shell (>10%), and 20 line-specific/cloud *bHLH*s ($\leq$10%) (Supplemental Data 7). On the basis of conservation levels, we adopted a systematic pangene nomenclature and renamed the 201 barley pangenome bHLH OGGs *HvbHLH.CR001–CR140* (core), *HvbHLH.SC141–SC152* (softcore), *HvbHLH.SH153–SH181* (shell), and *HvbHLH.CL182–CL201* (cloud). Within each category, the *bHLH* pangenes were named according to their genomic locations in increasing order (Supplemental Data 7).

Among the annotated genes, the number of core *bHLH*s ranged from 137 to 142 (greater than 140 because some core *bHLHs* were present in more than 1 copy) (Supplemental Data 7), and that of dispensable *bHLHs* ranged from 14 to 28 (Figure 1B). Among the newly predicted *bHLHs*, the number of core *bHLHs* ranged from 0 to 4 (average 1.15 across the 20 pangenomes), and that of dispensable *bHLHs* ranged from 4 to 10 (average 6.3) (Figure 1C). Three (*HvbHLH.CR137–CR139*) of the 140 core *bHLH*s were not annotated in the Morex reference genome (version 2) but were annotated in the remaining 19 varieties (Supplemental Data 7). A similar observation could also be made for the other barley genomes. These observations may reflect variations in genome annotation quality, highlighting the need for complete genome scanning for gene-family studies.

Among the 12 softcore barley *bHLH* pangenes, 1 softcore *bHLH* (*HvbHLH.SC152*) was not annotated in any genome but was newly predicted in this study. Three of the 12 softcore *bHLHs* were not annotated in Morex version 2. Among the 29 shell *bHLH* pangenes, 9 were not annotated in any genome but were newly predicted in this study. Five of these (*HvbHLH.SH162*, *HvbHLH.SH170*, *HvbHLH.SH177*, *HvbHLH.SH178*, and *HvbHLH.SH180*) were present in more than half (10–17) of the 20 barley genomes and thus warrant attention in future studies. Lastly, 7 of the 20 cloud *bHLH* pangenes were newly predicted, and the others were annotated in at least 1 barley genome. Taken together, these results highlight the clear advantage of the pangenome approach for gene-family screening and the discovery of novel gene members not present or annotated in a single reference genome.

Pangenome-based phylogenetic classification of the *bHLH* gene family in barley

To investigate the genuine evolutionary status of *bHLHs* in barley, particularly the dispensable *bHLH*s and the *bHLH*s absent in Morex, we performed a phylogenetic analysis of the 201 identified *bHLH* OGGs. For each OGG, the Morex *bHLH* gene was used as the representative sequence. For the 40 OGGs absent in Morex, the longest *bHLH* for each OGG in the remaining 19 barley genomes was used. Because of the highly divergent nature of the bHLH protein sequences, the conserved bHLH domain sequences of the 201 bHLH pangenes were used for phylogeny construction. *Oryza sativa* (subsp. *japonica*) (rice) *bHLHs* were used as references for subfamily classification. The 201 barley pangenes could be divided into 23 subfamilies (Figure 2; Table 1; Supplemental Data 7 and 8), 22 of which matched previous subfamily classifications in rice (Carretero-Paulet et al., 2010), suggesting that the *bHLH* subfamilies are generally conserved. No direct homolog for rice SF17 could be identified in barley on the basis of the bHLH domain sequences (Figure 2; Table 1). SF16 (6 in barley and 7 in rice) contained atypical *bHLH*s that lacked the DNA-binding motif. The remaining bHLH-domain-containing subfamily (called SF_like; Figure 2), which included 4 rice genes and 10 barley OGGs, was not included in the previous classification (Carretero-Paulet et al., 2010). Compared with rice *bHLH*s, 9 barley bHLH subfamilies (SF01, SF02, SF03, SF09, SF12, SF13, SF27, SF28, and SF_like) had higher gene numbers, and 13 subfamilies (SF04, SF05, SF07, SF08, SF10, SF11, SF15, SF16, SF23, SF24, SF25, SF26, and SF31) had lower gene numbers (Table 1; Supplemental Data 8). These variations may reflect the species-specific evolutionary profiles of *bHLHs* in barley and rice. In addition, one subfamily (SF14) had equal gene numbers in barley and rice (Table 1; Supplemental Data 8).

To compare the pangenome *bHLH* analyses with the reference-genome-based approach, the numbers of *bHLHs* from each subfamily present in Morex were counted and compared. Only 13 subfamilies (SF01, SF04, SF05, SF07, SF08, SF09, SF11, SF12, SF14, SF23, SF25, SF26, and SF31) had equal gene numbers between the barley pangenome and the reference genome (Table 1). By contrast, more members of the remaining 10 subfamilies (SF02, SF03, SF10, SF13, SF15, SF16, SF24, SF27, SF28, and SF_like) were identified in the pangenome than in the

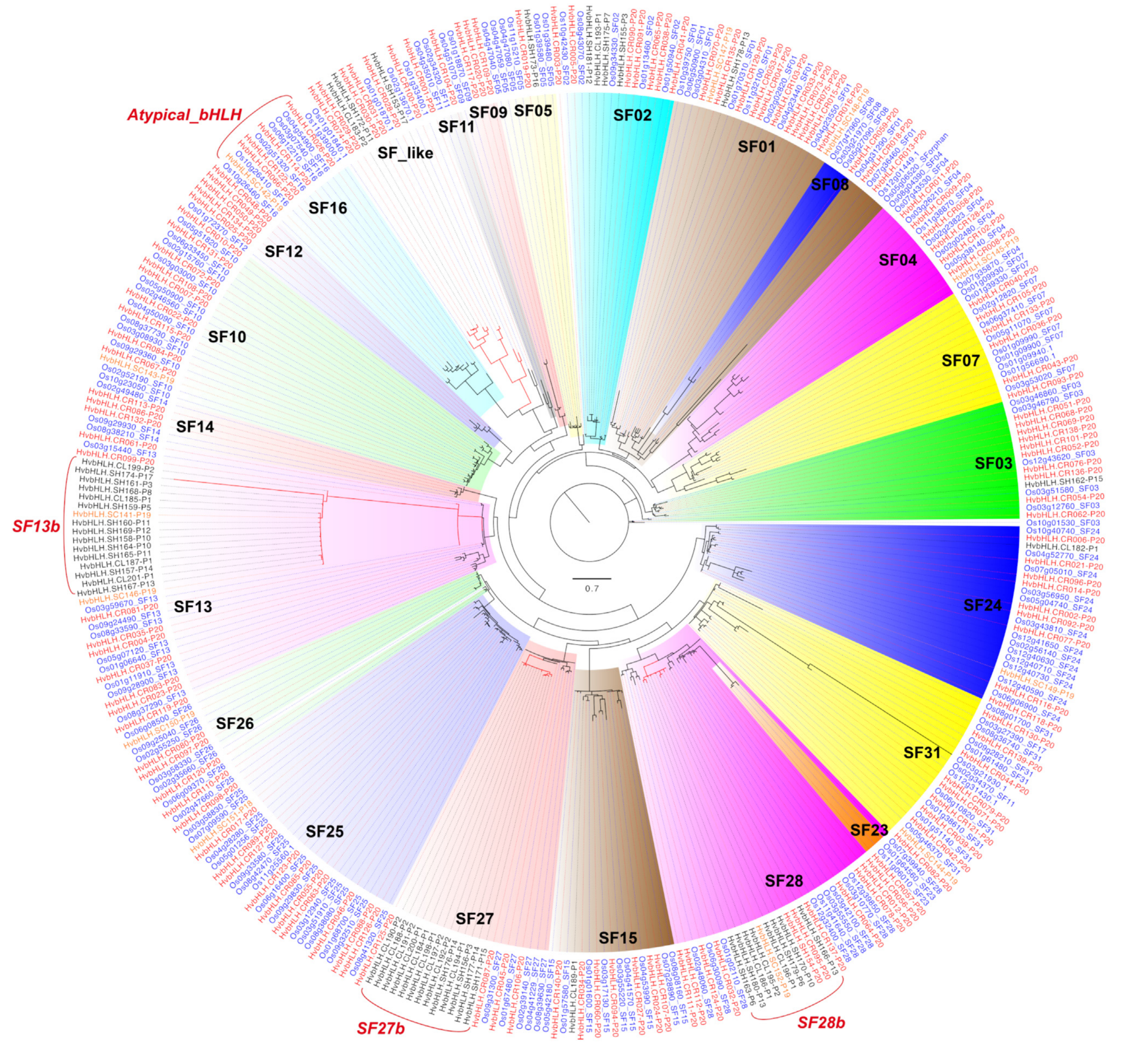

Figure 2. Phylogenetic classification of barley and rice *bHLHs*

The phylogeny was constructed from an aa sequence alignment of the bHLH domain. Representative *bHLH*s for 201 barley OGGs were included, using rice *bHLH*s (blue) as a reference. Barley *bHLH*s were classified into 3 categories: core (red), softcore (orange), and shell/cloud (black), on the basis of their conservation in 20 barley genomes. Subfamilies are highlighted in different colors and labeled accordingly. The branch for the SF_like subfamily is highlighted in red. The suffixes (P1–P20) in the barley *bHLH* pangene IDs indicate the number of barley lines containing that gene. For instance, P20 indicates that the gene is present in all 20 varieties. Three gene-expansion hotspots (SF13b, SF27b, SF28b) containing enriched dispensable *bHLHs* and atypical *bHLH*s are labeled correspondingly.

reference genome. The former 13 subfamilies (mainly core *bHLH*s) tended to be more conserved, whereas the latter 10 subfamilies contained more dispensable *bHLH*s (Figure 2).

Interestingly, the dispensable *bHLH*s were not distributed evenly among different subfamilies but were enriched in a few specific subfamilies, particularly in SF13, SF27, and SF28 but also in SF02 and SF_like (Figure 2). Within SF13, SF27, and SF28, the dispensable *bHLHs* tended to form sister clusters (SF13b, SF27b, and SF28b in Figure 2) and may represent genes under active duplication and selection. The enrichment of dispensable *bHLH*s in specific subfamilies suggests that expansion of the *bHLH* gene family in barley may be biased toward the evolution of specific biological functions and genetic adaptation to specific environmental conditions.

Analysis of protein motifs and gene structures

To further investigate the evolutionary relationships and expansion patterns of *bHLH*s in barley, we analyzed protein motifs

Subfamily	Rice	Morex	Panbarley	CoreSingle	withCNV	Characterized genes	Function	References
SF01	12	16	16	12	4	Os02g02820, Os07g36460, Os03g04310, Os10g39750, Os04g23550, Os04g31290; HORVU.MOREX.r2.4HG0340630.1	regulate strategy II iron acquisition, early anther development, phosphate starvation response, pollen development; seed priming-induced drought tolerance in barley	s1–s6
SF02	5	8	10	6	4	Os10g42430/OsMYC2, AtNIG1, AtMYC2	regulate the mechanism of grass spikelet development via jasmonic acid; bind with calcium and enhance salt tolerance; enhance proline level and salt tolerance	s7, s47, s48
SF03	5	10	11	8	3	At2g22770/At020NAI1	regulate the formation of an endoplasmic reticulum (ER)-derived structure, the ER body	s8
SF04	8	7	7	6	1	Os03g26210/OsIRO3, Os07g43530, At5g54680/AtILR3, At1g51070/AtbHLH115	regulate jasmonate (JA) signaling and Fe-deficiency response; regulate iron balance	s9, s10, s53, s54
SF05	6	3	3	2	1	At1g63650/At002EGL, At4g00480/At012MYC, At4g09820/At042TT8; HORVU.MOREX.r2.4HG0326660.1	specify epidermal cell fate in the *Arabidopsis* root; regulate trichome and root hair initiation as well as flavonoid accumulation; regulate flavonoid biosynthesis in barley grain	s11–s14
SF07	10	6	6	6	0	Os03g53020, OsbHLH148	related to drought tolerance via JA signaling; involved in the JA pathway and increased drought tolerance	s15, s52
SF08	3	1	1	1	0	–	–	–
SF09	2	3	3	3	0	Os04g51070, Os01g18870	regulate tapetal programmed cell death, pollen development, and the differentiation, morphogenesis, and degradation of anther somatic cell layers	s5, s16, s17
SF10	12	9	10	9	1	At3g06120/At045MUTE, At3g24140/At097FM, At5g53210/At098SPC	involved in stomatal development	s18–s21

Table 1. Distribution of *bHLHs* in rice and barley and functionally characterized *bHLH*s based on literature review.

(Continued on next page)

Table 1. *Continued*

Subfamily	Rice	Morex	Panbarley	CoreSingle	withCNV	Characterized genes	Function	References
SF11	3	1	1	1	0	At1g49770/At095ZOU	involved in endosperm development; regulate differentiation, morphogenesis, and degradation of anther somatic cell layers; regulate tapetal programmed cell death and pollen development	s22, s23
SF12	1	5	5	5	0	Os01g72370; HORVU.MOREX.r2.3HG0268450.1; AtbHLH38	involved in the regulation of gene expression under Fe-deficient conditions; respond to copper deficiency	s24, s51
SF13	9	10	25	8	17	–	–	–
SF14	3	3	3	3	0	At5g08130/At046BIM1, At1g69010/At102BIM, At5g38860/At141BIM3	involved in brassinosteroid signaling	s25
SF15	9	7	8	7	1	At2g31730/At154ERP, TabHLH123	similar to ethylene-regulated protein LeER33; an essential regulator of crown root initiation	s26, s41
SF16	7	4	5	4	1	Os04g54900, Os11g39000.1, Os03g07540, Os06g12210, Os02g51320, Os10g26460 (atypical *bHLHs*)	regulate cell elongation, plant development, and grain length; involved in brassinosteroid signaling and lamina joint bending	s27–s29
SF17	2	0	0	0	0	Os03g27390	regulate male fertility at different temperatures	s39, s40
SF23	2	1	1	0	1	At2g31280/At155CPu; At2g27230/At156LHW	regulate early xylem development downstream of auxin; promote procambial-cell-specific proliferation through cytokinin response	s30, s31
SF24	13	10	11	9	2	Os03g56950, Os05g04740	regulate internode elongation and induce a morphological response to drought stress	s27, s32
SF25	16	13	13	12	1	Os03g58830, Os07g09590, AT1G25330; ZmbHLH55	regulate JA-induced gene expression and root cell elongation; involved in ABA biosynthesis and improve salt tolerance	s33, s44, s50
SF26	6	5	5	4	1	At4g02590/At059UN12; HORVU.MOREX.r2.7HG0546900.1, TabHLH1, OsbHLH98	involved in female gametophyte development; root epidermal cell differentiation; cause a lack of root hairs in barley; reduce leaf water loss rate and increase proline and soluble sugar contents in wheat; negative regulation of leaf angle in rice	s34, s35, s42, s45

(*Continued on next page*)

 © 2024 The Authors.

Subfamily	Rice	Morex	Panbarley	CoreSingle	withCNV	Characterized genes	Function	References
SF27	5	7	17	4	13	At1g51140/AtbHLH122	regulate drought and osmotic stress resistance	s49
SF28	10	13	20	11	9	Os12g32400; SbbHLH85	regulate iron distribution between roots and shoots; increase the number and length of root hairs; increase salt resistance via sodium ion absorption	s36, s46
SF31	12	10	10	9	1	Os01g61480, Os05g46370, OsLAX	regulate axillary meristem formation and flowering; regulate the formation of the axillary bud primordium	s37, s38, s43
SF_like	4	8	10	7	3	–	–	–
Total	163	161	201	137	64	–	–	–

Table 1. Continued
Subfamily classification is based on the phylogenetic analysis in Figure 2. For each subfamily, the numbers of *bHLHs* in rice, pan_barley (1 sequence for each orthologous group), and Morex version 2 were counted. Gene IDs for previously characterized *bHLHs* and their reported functions were retrieved from the corresponding references (see Supplemental Data S1).

and gene structures of the *bHLH* OGGs. Overall, we observed that bHLHs with close evolutionary relationships had similar protein motifs (Supplemental Figure 1A) and gene structures (Supplemental Figure 1B). bHLHs within the same subfamily tended to have comparable protein lengths, but their gene lengths could vary significantly, mainly because of differences in intron regions. The protein sequence lengths of bHLHs in some subfamilies, such as SF01, SF02, SF05, SF07, SF24, SF25, and SF31, also varied greatly, mainly owing to protein regions outside the bHLH domain. Motif 1 and motif 2 belonged to the bHLH domain (Supplemental Data 9) and were generally conserved, except in SF13b, in which motif 1 was replaced with motif 7. The latter motif was also recognized as the bHLH domain, albeit with lower significance. In addition, 6 newly predicted bHLHs in SF28b (4), SF31 (1), and SF01 (1) contained only partial motif 1 or motif 2 and were not annotated (Supplemental Figure 1A). Three of these 6 newly predicted partial bHLHs (HLHg324, 10350_HLHg168, and HLHg063) were core bHLHs. In addition, 10 bHLHs from SF03 (2), SF08 (1), SF24 (1), SF27b (1), and SF28b (5) contained either motif 1 or motif 2 only (Supplemental Figure 1A). Out of the 21 newly predicted representative bHLHs (Supplemental Data 7), 14 were found to contain complete bHLH domains (Supplemental Figure 1A). The functions of these newly predicted bHLHs will need to be investigated in future studies. Most of the dispensable bHLHs were found to share similar protein-motif profiles with the core bHLHs in the same subfamily.

In contrast to motif 1 and motif 2, the other motifs tended to be subfamily specific. Motif 10 was present mainly in the upper half of the phylogenetic tree but missing in the lower half. Motif 9 was strictly conserved in SF25, SF26, and SF27 and partially conserved in SF01, SF05, SF07, and SF28. The position of motif 9 relative to motif 1 or 2 could change depending on the specific gene. Motif 8 is strictly conserved in SF12 and SF_like and is present in 2 additional bHLHs from SF10 and SF13. The exchange of these motifs across different subfamilies may imply potential protein recombination during bHLH evolution. Interestingly, motifs 7, 4, 3, 6, and 5 seem to be unique to and highly conserved in the dispensable bHLHs of subfamily SF13b and may thus represent a novel type of SF13 under active evolution. In contrast to the unique motifs in SF13b, the dispensable bHLHs in SF27b seemed to be shorter and truncated (missing motif 9) versions of SF27 and were also highly conserved. By contrast, the dispensable SF28bs were more likely to have a partial bHLH domain and were less conserved. These dispensable bHLHs deserve special attention in future studies of gene function.

In terms of gene structure, exon numbers in the barley *bHLH*s ranged from 1 to 12 (Supplemental Figure 1B). Within the same *bHLH* subfamily, the exons were generally conserved, whereas the number and length of introns could vary significantly, potentially leading to transcriptional divergence. Interestingly, all except 2 of the dispensable *bHLH*s in SF13b had a single exon and may therefore be partial gene sequences (lacking annotation outside of the bHLH domain region because of the highly divergent nature of the remaining sequence). Additional data from *de novo* transcriptome assemblies should be used to identify the full sequences and gene structures of these potentially partial bHLHs.

Analysis of CNVs, synteny, gene duplications, and transposon elements

For pangenome-based gene-family analyses, CNVs (including gPAVs) for each *bHLH* pangene provide important biological insights into gene and plant evolution. To assess CNV in *bHLHs* across the barley pangenome, we counted the gene copy number of each pangene. Overall, the number of genes in each pangene ranged from 1 (a unique *bHLH* in a specific barley line) to 37 (present in multiple copies in some barley genomes). On the basis of gene copy-number pattern, the 201 bHLH pangenes included 137 single-copy core bHLHs (Table 1; Supplemental Data 10). These core single-copy bHLHs covered all 23 bHLH subfamilies except for SF23 (*HvbHLH.SH179*), which was identified in only 6 of the 20 barley pangenomes. The other 64 bHLH pangenes with CNVs (see Figure 4) could be divided into 3 categories: multiple-copy core *bHLHs*, multiple-copy dispensable *bHLHs*, and single-copy dispensable *bHLHs*. Although most core *bHLHs* were strictly conserved as a single copy, 3 core *bHLHs* (SF01: *HvbHLH.CR135*; SF03: *HvbHLH.CR051*, *HvbHLH.CR101*) had 2 copies in some varieties (Figure 3). Eight *bHLHs* (SF01: *HvbHLH.SH178*; SF02: *HvbHLH.SH181*; SF13b: *HvbHLH.SC141*, *HvbHLH.SC146*, *HvbHLH.SH158*; SF27b: *HvbHLH.SH171*, *HvbHLH.CL198*; SF28b: *HvbHLH.SC152*) were identified as dispensable *bHLHs* that were present in 2–3 copies in some genomes but were absent in others. These multiple-copy dispensable *bHLHs* may represent *bHLHs* under active duplication, playing an important role in micro-environmental adaptation. Indeed, this type of *bHLH* contained the most highly expanded *bHLH* OGGs (SF13b: *HvbHLH.SC146*; SF28b: *HvbHLH.SC152*) (Figure 3). By contrast, the remaining single-copy dispensable *bHLHs* were relatively stable and may not be under active duplication. In addition, most line-specific *bHLHs* were also present in single-copy form (Figure 3).

To explore the mechanism of *bHLH* expansion in barley, the gene duplication types and syntenic relationships of the *bHLHs* were characterized. The genomic locations of 151 annotated and 10 newly predicted *bHLHs* in Morex are shown in Supplemental Figure 2. Chromosomes 1H and 6H had the lowest numbers of *bHLHs*, whereas the other chromosomes contained more *bHLHs*. A total of 38, 13, 6, and 94 *bHLHs* were identified as whole genome/segmental (purple), tandem (green), proximal (pink), and dispersed (blue) duplicates, respectively, suggesting that *bHLHs* in barley have mainly expanded via dispersed duplication.

To assess whether the conservation level of *bHLHs* in the barley pangenomes might be related to gene duplication type, the *bHLH* gene duplication types (whole-genome duplication [WGD]/segmental duplication, dispersed duplication, tandem duplication, proximal duplication, dispersed duplication, and singleton) for each conservation category (core, softcore, shell, and cloud) were counted and analyzed in the 20 barley pangenomes. The results (Figure 4A; Supplemental Data 11) showed that for the 3411 *bHLHs* identified in the 20 barley genomes, dispersed duplication was the most common mechanism (58.8%) contributing to *bHLH* expansion, followed by WGD/segmental duplication (18.3%). Tandem duplicates, proximal duplicates, and singletons accounted for 6.3%, 7.9%, and 8.8% of the *bHLHs*, respectively. The majority of *bHLHs* were identified as core *bHLHs*, accounting for 82.5%, followed by shell (9.2%) and softcore (7.4%) and a small number (0.9%) of line-specific/cloud *bHLHs*. In terms of gene duplication type, core and softcore had a similar ratio of WGD/segmental to dispersed duplications, consistent with their conservation level (Figure 4A). By contrast, WGD/segmental duplication was rare in shell and cloud bHLHs. We found that dispersed duplication was more dominant in shell bHLHs and that tandem duplication was rarely observed in softcore bHLHs. WGD/segmental duplication rarely contributed to shell and cloud bHLHs. Intriguingly, tandem duplication occurred predominantly in core genes but not in softcore bHLHs. Proximal-duplication bHLHs did not show any clear correlation with duplication type. These data provide new insights into the molecular mechanisms underlying CNVs in the bHLHs.

Transposon elements (TEs) are a major driver of gene duplication and CNV in plant genomes (Ma et al., 2023). To further investigate the potential genetic mechanisms underlying *bHLH* CNVs in barley, we predicted TEs in the 20 barley pangenome assemblies and searched for TEs that occurred within 2 kb upstream/downstream (including the gene region) of the *bHLHs* identified in this study. Only intact TEs were considered. The results (Figure 4B; Supplemental Data 12) showed that 1707 (50%) of the 3411 *bHLHs* in the barley pangenome contained intact TEs within 2 kb upstream/downstream of the gene. We noted that 464 *bHLHs* contained more than 1 (2 to 9) intact TEs within the 2-kb upstream/downstream regions. DNA transposons (TcMar, PIF-Harbinger, MULE, hAT, CMC-EnSpm) accounted for the majority (~75%) of TEs identified within or close to *bHLHs*, and RNA-mediated retrotransposons (Helitron, Copia, Gypsy, SINE, LINE) accounted for around 25%. For *bHLH* pangenes with CNVs, an average of 1.48 TEs were identified per gene, significantly (p = 0.0145) more than the 1.36 TEs per gene identified for *bHLHs* without CNVs, suggesting a potential role for TEs in CNVs (Figure 4C). In terms of *bHLH* subfamilies, although the numbers of TEs identified were generally proportional to the number of core *bHLHs* in each subfamily (Figure 4D; Table 1; Supplemental Data 12), a few exceptions were observed. For example, SF25 contained the largest number of core *bHLHs* but only accounted for 6.3% of the TEs, in contrast to SF01 (14.0% of the TEs) and SF28 (9.8% of the TEs). Notably, SF13 contained a moderate number of core *bHLHs* but accounted for 9.5% of the TEs, clearly owing to the marked expansion of SF13b identified in this study. Indeed, further examination showed that SF13b accounted for 28% of the TEs in SF13. The same observation could be made for SF27b, which accounted for 48.8% of the TEs in SF27. However, this did not seem to be the case for SF28b, which accounted for only 9.5% of the TEs in the SF28 subfamily. These results suggest that the potential contribution of TEs to *bHLH* family expansion may vary across different subfamilies.

Analyses of natural selection

The ratio (ω) of non-synonymous (*Ka*) to synonymous (*Ks*) substitutions is used to assess gene selection pressure, with $\omega < 1$, $\omega = 1$, and $\omega > 1$ indicating purifying, neutral, and positive selection, respectively. To assess the natural selection pressure acting on barley *bHLHs*, values of *Ka*, *Ks*, and *Ka/Ks* were calculated for each OGG (only those present in Morex were included). When selection pressures on core and dispensable *bHLHs* were compared,

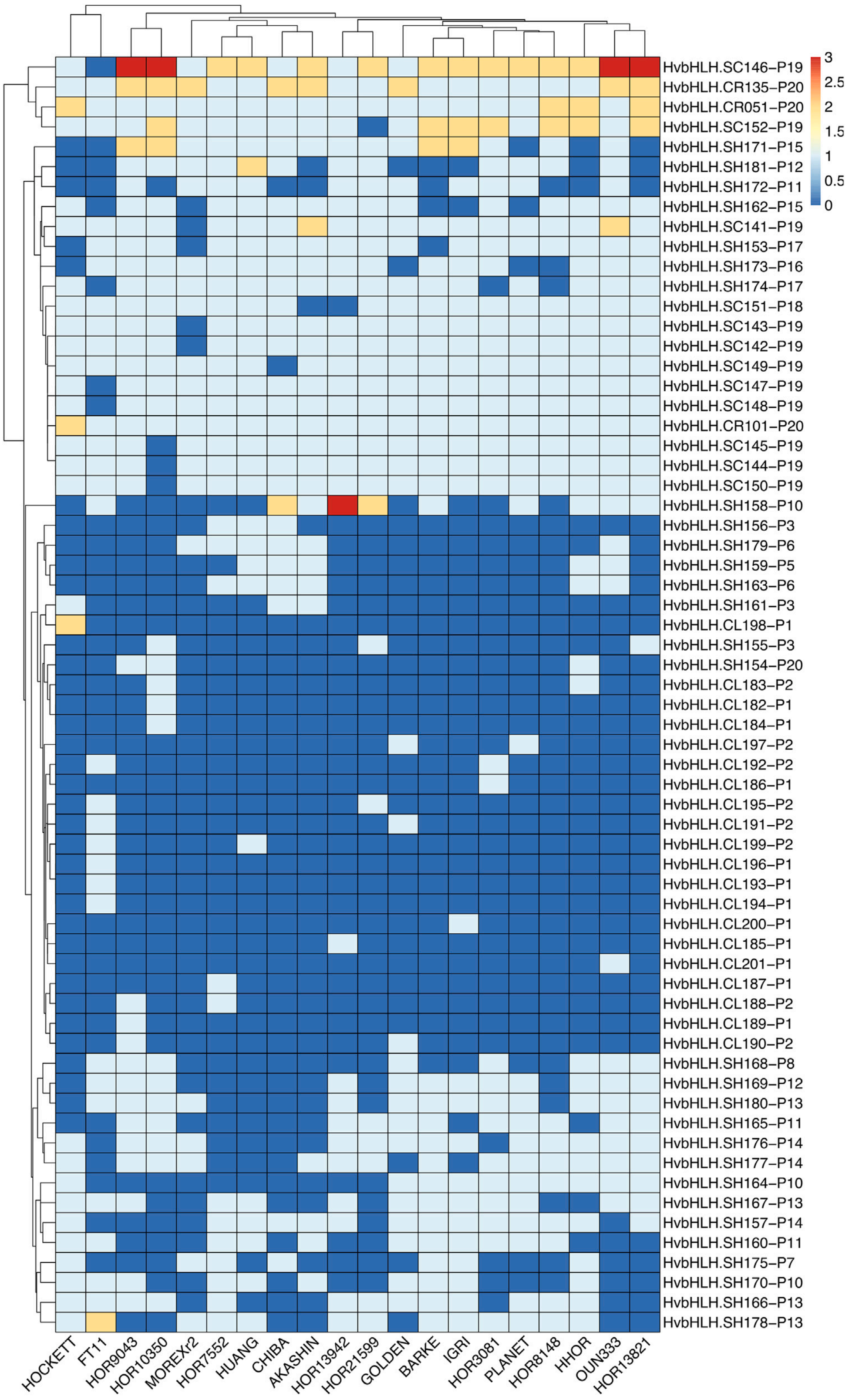

Figure 3. Heatmap displaying CNVs of *bHLH*s in the barley pangenome

The gene copy numbers for the 64 barley *bHLH* pangenes with CNVs were used for heatmap construction. Clustering based on CNV was implemented at both the gene and variety levels. The complete gene copy matrix for 201 pangenes is provided in Supplemental Data 10.

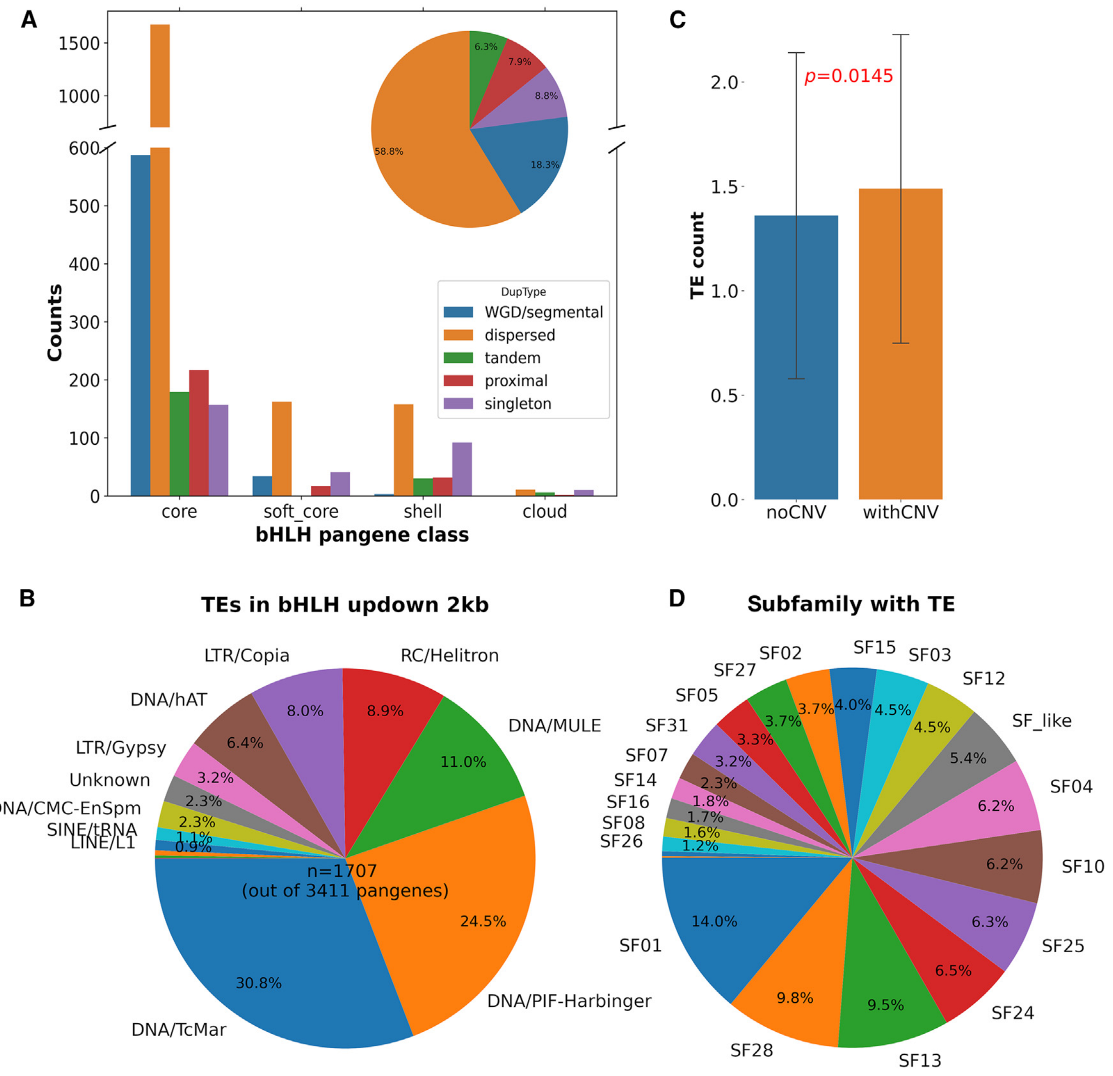

Figure 4. Gene-duplication and TEs analyses of the barley *bHLHs*

(A) Distribution of different duplication types for core, softcore, shell, and cloud *bHLHs*.

(**B**) Distribution of different types of TEs identified in the 2-kb upstream to 2-kb downstream region of *bHLHs*.

(**C**) Number of TEs identified in *bHLHs* with (n = 256) and without (n = 1451) CNVs. The p values were calculated using 2-way t tests.

(**D**) Distribution of TEs across different *bHLH* subfamilies.

See Supplemental Data 11 and 12 for detailed data.

dispensable *bHLHs* had significantly (p = 8.721e−7) higher median *Ka* values (Figure 5A, left) than core *bHLHs*. Their median *Ks* values were more similar, although statistically different at a much lower significance level (p = 1.301e−7) (Figure 5A, center). Dispensable *bHLHs* had significantly (p = 1.560e−2) higher *Ka/Ks* values than core *bHLHs* (Figure 5A, right), and the *Ka/Ks* values for dispensable *bHLHs* were also more divergent than those for core *bHLHs*. These observations suggest that dispensable *bHLHs* have generally experienced relaxed selection pressure compared with core *bHLHs*, which appear to have been under stricter constraints of purifying selection.

Next, the selection pressures on different subfamilies were calculated and compared. The average *Ka* values across different *bHLH* subfamilies ranged from 0 to 0.046 and were much lower than the average *Ks* values, which ranged from 0.006 to 0.159 (Figure 5B). Subfamilies SF01, SF02, and SF04 had higher *Ka* and *Ks* values, indicating a higher mutation rate in these 3 subfamilies, which may be associated with relaxed selection pressure. The average *Ka/Ks* ratios for individual subfamilies ranged from 0.01 (SF16) to 0.5 (SF09), implying generally purifying but also varied selection pressures across different *bHLH* subfamilies. The highest *Ka/Ks* values (0.4–0.5) were observed for SF24, SF09, SF03, and SF12. However, the overall *Ka* and *Ks* values for these 4 subfamilies remained relatively low, suggesting that these 4 subfamilies may be affected by positive selection rather than relaxed selection pressure.

Lastly, the detailed selection-pressure profile for each *bHLH* was visualized and examined (Supplemental Figure 3). Overall, dispensable *bHLHs* clearly had higher *Ka* and *Ks* values, consistent with the hypothesis that disposable *bHLHs* may be

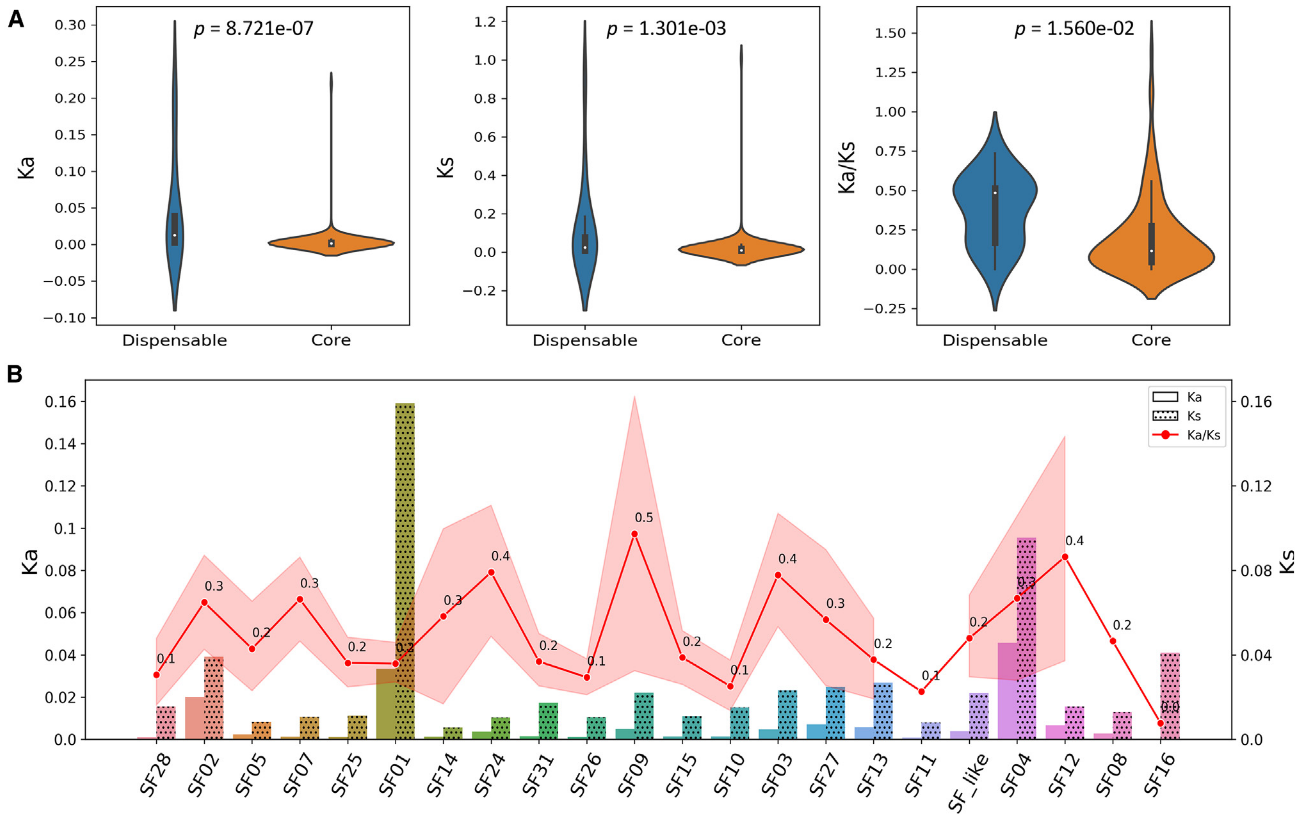

Figure 5. Analysis of natural selection on barley *bHLHs* at the pangenome level

(A) Comparison of *Ka*, *Ks*, and *Ka/Ks* values between core (n = 137) and dispensable (n = 18) *bHLH*s. White dots indicate median values; p values were calculated using 2-way t tests.

(**B**) Comparison of *Ka*, *Ks*, and *Ka/Ks* values across different subfamilies. Mean *Ka/Ks* values are labeled above each dot. The SD error band (red) for *Ka/Ks* indicates the 85% confidence interval. Subfamilies without error band coverage contain only a single gene or have had exceptional selection values removed.

See Supplemental Data 13 for detailed calculation data.

under less functional constraint. Although most core *bHLH* genes had low *Ka*, *Ks*, and *Ka/Ks* values, consistent with strictly conserved functions, the highest *Ka/Ks* (but not *Ka* or *Ks*) values generally occurred in core *bHLH*s. This observation suggests that core *bHLH*s are more likely than dispensable *bHLHs* to be affected by positive natural selection, whereas higher *Ka/Ks* values of dispensable *bHLH*s are more likely to be associated with relaxed natural selection. In summary, analyses of natural selection demonstrated that mutation rates and natural-selection pressures tended to vary not only between core and dispensable *bHLHs* but also across different *bHLH* subfamilies. Further studies are needed to examine whether these variations may be related to their specific biological functions.

Transcriptome analyses

To characterize the transcriptional profiles of *bHLH*s, we analyzed recently released pantranscriptome sequencing data (Waugh et al., 2024) for 5 tissues (shoot, root, inflorescence, coleoptile, and caryopsis) in 20 barley genomes. First, we extracted expression data for all the newly predicted *bHLHs* in each variety and found that more than half of the newly predicted *bHLHs* were actively transcribed in the 5 tissues examined (Supplemental Data 14). This provided direct support for the validity of our bHLH gene-prediction results. Second, values of transcripts per kilobase million (TPM) for the *bHLH* pangenes were extracted and their mean and SD calculated across the 20 barley genomes. The *bHLHs* were clustered on the basis of their expression patterns, and the clustering results were compared with the gene phylogeny (Figure 6A). The gene expression levels and their SDs in the 5 tissues across the 20 barley genomes were visualized as heatmaps in Figure 6B and 6C, respectively. Clustering by gene expression revealed 3 major clusters (C1–C3 in Figure 6, right tree) with distinct transcriptional profiles. Overall, the C1 cluster included *bHLH genes* highly expressed in most tissues, C2 included tissue-specific genes, and C3 included genes highly expressed in 2–4 tissues. Most of the annotated *bHLH*s in the 20 genomes were actively expressed in at least 1 tissue, consistent with their conservation as core *bHLH* genes. Analysis of relative SDs across the 20 barley genomes (Figure 6C) revealed a clear negative correlation with the gene expression heatmap—in other words, genes with higher expression levels showed lower variation in expression across different barley genomes, whereas genes with weak expression tended to have more variable expression across different barley lines.

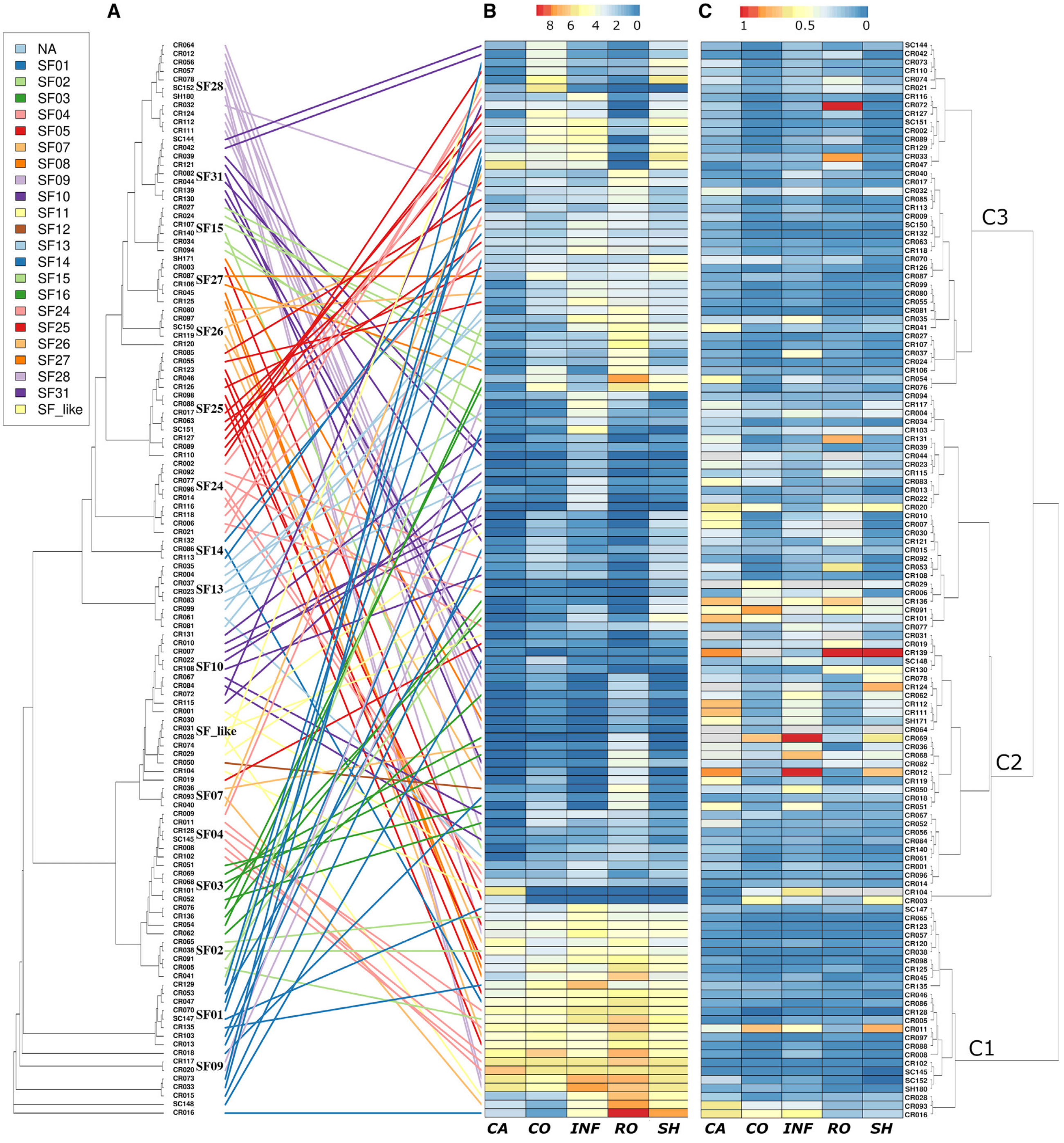

Figure 6. Pantranscriptome analyses of *bHLH*s in barley

Comparison of gene phylogeny with expression profile clustering **(A)**, heatmap of mean expression **(B)**, and heatmap of gene-expression relative SD values in the pangenome **(C)** of annotated *bHLH*s from 20 barley accessions in 5 barley tissues (CA, caryopsis; CO, coleoptile; INF, inflorescence; RO, root; SH, shoot). Transcriptome data were obtained from a recent study (Waugh et al., 2024). Gene expression values were normalized using $\log_2$(TPM+1) transformation. Relative SD was calculated as the SD divided by the mean TPM, followed by $\log_2$(std+1) transformation. The gene phylogeny (left tree) was compared to the gene expression clustering cladogram (right tree) by linking lines (bHLH subfamilies highlighted in different colors). Three major *bHLH* clusters (C1–C3 at right) with distinct transcriptional patterns are highlighted and labeled.

To characterize the gene pantranscriptome profiles of different barley *bHLH* subfamilies, the gene transcriptional profiles and their clustering patterns (Figure 6, right tree) were compared (Figure 6A, linking lines) with the *bHLH* gene phylogeny (Figure 6, left tree). When viewed from right to left, the mixture of lines with different colors reveals that gene clusters with similar expression profiles were generally derived from multiple subfamilies (Figure 6A). For example, group C1, whose

members were universally highly expressed, contained *bHLH*s from subfamilies SF01, SF02, SF04, SF25, SF26, and SF27. This pattern applied to all major gene transcription clusters (Figure 6, right tree). Therefore, the linking lines and their mixed colors to the right in Figure 6A provide a direct visual representation of the possibility that *bHLH*s from different subfamilies may function coordinately in a common biological process. However, when viewed from left to right, the variations in line angles for genes in the same subfamily in Figure 6A provide a direct estimate of the transcriptional divergence of each subfamily. Our results show that most *bHLH* subfamilies generally contain members with divergent expression profiles across different tissues. In particular, *bHLH* subfamilies with the most divergent expression profiles included SF31, SF15, SF27, SF26, SF25, SF14, SF_like, and SF01 (top to bottom in Figure 6, left tree). These results support the idea that transcriptional divergence is a common phenomenon during gene evolution. We also noticed that neighboring gene pairs (Figure 6, left tree), which represent recently duplicated gene pairs, could display either similar expression profiles (similar line angles, such as the first 2 genes in SF31) or highly divergent expression profiles (varied line angles, such as SF07, SF14, and SF26). In addition, the 7 *bHLH*s in SF04 were clearly separated into 2 groups: 4 universally highly expressed genes and 2 genes highly expressed in the inflorescence (Figure 6, left tree). We also want to highlight 3 bHLHs (Figure 6A, *HvbHLH.SH180* and *HvbHLH.SC152* in SF28, *HvbHLH.CR139* in SF31) that were not annotated in Morex version 2. Both *HvbHLH.SH180* and *HvbHLH.SC152* were highly expressed in all 5 tissues, and *HvbHLH.CR139* was moderately expressed in all tissues except the coleoptile (Figure 6B).

In summary, Figure 6 provides a template for how pantranscriptome data can be integrated with pangenome-based gene evolutionary analyses to quickly and effectively present and interpret gene-family evolution in the pangenome era.

DISCUSSION

In this study, we proposed an innovative pangenome-wide approach for gene-family characterization, together with integrated pantranscriptome analyses. Instead of identifying *bHLH*s in a single reference genome, we scanned bHLH-encoding genes in 20 published barley genome assemblies (Jayakodi et al., 2020) and classified them into different OGGs. We used a systematic nomenclature and renamed the OGGs as pangenes: *HvbHLH.CR001–CR140* (140 core), *HvbHLH.SC141–SC152* (12 softcore), *HvbHLH.SH153–SH181* (29 shell), and *HvbHLH.CL182–CL201* (20 cloud). Representative sequences from these *bHLH* OGGs were used for downstream core/dispensable classification and phylogenetic, gene-structure, CNV, natural selection pressure, and pantranscriptomic analyses. This approach provided novel insights into *bHLH* gene evolution that would not be accessible if a single reference genome were used. We recommend this as a common naming system for future pangenome-based gene-family analyses. Furthermore, through the integration of recently released barley pantranscriptome data (Waugh et al., 2024), we provided a simple template (Figure 6) for the combination of pangenome gene-family analyses with pantranscriptome data that enables the effective visualization of transcriptional divergence or convergence within or across different subfamily members. Our integrated pangenome- and pantranscriptome-based gene-family characterization overcomes the limitations of traditional reference-genome-based methods and can describe the genuine evolutionary status of a *bHLH* gene family in barley from multiple, previously untapped perspectives. We expect that this study will inspire similar analyses of many other gene families and species.

Pangenome OGGs vs. reference genome genes

To date, most gene-family characterization studies have been performed only on reference genomes, with the gene content of a single genome used to represent an entire species. For example, we found 65 publications, spanning approximately 53 different species, in which genome-wide identification of *bHLHs* was performed, all of which were based on gene scanning in reference genomes (Supplemental Data 1). One clear limitation of these reference-genome-based studies is that they cannot identify *bHLH*s absent in the reference genome but conserved in other lines in the population. Recently published pangenome data have revealed that genetic variations such as gPAVs or CNVs are prevalent in the populations of various species (Bayer et al., 2020; Jayakodi et al., 2020). Regarding the underlying genetic causes of CNVs, TEs are known as important contributors to new gene duplication (Ma et al., 2023). In this study, we found that *bHLH*s with CNVs had significantly more TEs than those without CNVs, supporting TEs as potential contributors to *bHLH* CNVs. This is the first evidence of links between TEs and CNVs at the pangenome level. In addition, we observed that dispersed duplicates are the dominant duplicate type in barley *bHLHs*, which corroborates the fact that TEs account for 88.4% of the barley genome and lead to small-scale gene duplications. Dispersed duplication was more common in shell *bHLH*s, whereas WGD/segmental duplication rarely contributed to shell and cloud *bHLH*s. This implies an apparent correlation between the scale of gene duplication and the level of gene conservation, consistent with previous findings. Depending on the particular gene function, the presence of PAVs and CNVs in the pangenomes of crop species may be associated with various stress tolerances (Maron et al., 2013; Sarkar et al., 2019; Schiessl et al., 2019), which may have agronomic consequences and are important for crop improvement. In this study, we took gPAVs and CNVs into consideration and attempted to characterize the complete gene-family profile of *bHLHs* in barley at the pangenome level. Our analyses have several advantages over traditional reference-genome-based studies: First, pangenome OGGs cover *bHLH*s that are absent in reference genomes and reduce genome bias. For example, we found 40 *bHLH* OGGs that were absent in Morex, including 3 *bHLH*s that were strictly conserved in all other barley lines. The mutual validation of the newly predicted *bHLHs* in Morex versions 2 and 3 not only provided direct support for the reliability of our genome-scanning approach but also highlighted the genome annotation bias that may lead to different gene numbers and conclusions. These observations imply the prevalence of gPAVs and CNVs for *bHLH*s in barley populations and highlight the need for a pangenome to enable accurate gene-family analysis. Second, pangenome OGGs enable the

identification of core and dispensable *bHLH*s, which has important biological implications for gene evolution. For example, 137 and 14 of the 151 annotated bHLHs in Morex were found to be core and dispensable, respectively, which provides critical insights into gene function. Third, pangenome OGGs enable the calculation of natural selection pressure acting on each *bHLH*. In particular, we found that dispensable *bHLH*s were under relaxed selection pressure compared with core *bHLH*s, consistent with the findings of our recent pangenome gene-retention rate study (Jia et al., 2023) and making good biological sense. The variation in selection pressure between core and dispensable *bHLH*s also provides important information on gene evolution.

The total number (201) of OGGs was much higher than the *bHLH* gene numbers in individual genomes, a finding that is apparently caused by the presence of gPAVs. Even if we subtract the cloud *bHLH*s present in only 1 or 2 barley lines (20 in total), the remaining number of OGGs (181) is still higher than the number of *bHLH*s (161) identified in Morex, clearly highlighting the limitation of using a single reference genome for bHLH gene-family analyses in barley. We speculate that similar observations would be made for *bHLH*s in other species if their pangenomes were searched, which would enable the identification of new *bHLH* members that have been missed in previous studies. The number of distinct OGGs compared with the gene number in each genome provides a direct estimate of gPAVs for *bHLH*s in the barley pangenome. A recent study identified only 141 *bHLH*s in the barley reference genome (Morex version 1) (Ke et al., 2020), all of which were present in our gene list. The identification of extra *bHLH*s in Morex may be attributed to differences in genome annotation (Morex version 2 in this study), gene scanning, and selection criteria. We noticed that the aa sequences of some newly identified bHLHs are quite short (40–50 aa). We reason that this is due to the well-known nature of the bHLH TF family, in which only the bHLH domain motifs are conserved and the protein sequences outside the bHLH domain usually exhibit extremely low (if any) sequence conservation (Carretero-Paulet et al., 2010). In this study, we aimed to fully document the complete bHLH content by whole-genome scanning based on both sequence homology blast and Pfam domain hmmscan, an innovative algorithm implemented by BITACORA (Vizueta et al., 2020). Because protein sequences outside the bHLH domain rarely exhibit conservation, for some novel or less-characterized bHLHs (in this case, some newly identified OGGs), we expect that the current method will identify only the highly conserved bHLH domain sequences but not the complete protein sequences. We found direct evidence in support of this hypothesis from the mutual validation between Morex versions 2 and 3. Future studies should aim to determine the complete sequences of the partial bHLHs identified in this study. Nonetheless, several studies have identified functional *bHLH*s with partial bHLH domains, including atypical *bHLH*s without the DNA-binding motif (Zhang et al., 2009; Carretero-Paulet et al., 2010; Zheng et al., 2017). In addition to the atypical *bHLH*s, *bHLH*s with partial bHLH domains have also commonly been identified (Carretero-Paulet et al., 2010; Zheng et al., 2017; Ke et al., 2020; Zhou et al., 2020; Fan et al., 2021; Shen et al., 2021; Sun et al., 2022a). On the basis of pantranscriptome data, we found that more than half of the newly predicted bHLHs were actively transcribed in the 5 tissues examined, providing direct support for the validity of our gene-prediction results. *bHLH*s that were not expressed in the tissues examined in this study may still be transcribed and perform a function in specific combination(s) of tissues and environmental conditions. Therefore, it is reasonable to retain these truncated *bHLH*s for future gene functional analyses. Among monocot crop species, 146, 167, and 208 putative *bHLH*s have been identified in the reference genomes of *Brachypodium distachyon* (Niu et al., 2017), rice (Li et al., 2006), and maize (Zhang et al., 2018), respectively. We speculate that these gene numbers will also change significantly when the corresponding pangenomes are searched. Given the significantly higher number of OGGs than of *bHLH* genes in the barley reference genome, it is likely that comparative pangenome-based gene-family analyses will lead to the identification of new bHLH lineages or subfamilies, as was the case with SF13b, SF27b, and SF28b. Further study is needed to determine whether these dispensable *bHLH*s are also conserved in closely related species.

The biological implications of core and dispensable genes for *bHLH* evolution

The idea of core and dispensable genes emerged along with the publication of pangenome data for microbial organisms (Tettelin et al., 2005; Vernikos et al., 2015) and has recently been adopted for pangenome studies in plants (Gao et al., 2019; Bayer et al., 2020). Identifying core and dispensable genes has been suggested to have important biological implications for gene function (Della Coletta et al., 2021). Despite the widespread description of core and dispensable genes in previous pangenome studies, most of these analyses have stayed at the whole-genome level (Gao et al., 2019; Bayer et al., 2020). Only a limited number of studies (Van de Weyer et al., 2019; Sun et al., 2022b) have applied the idea of core and dispensable genes to a specific gene family. In this study, we classified barley *bHLH*s into core and dispensable categories on the basis of their conservation levels in the barley pangenome. This critical information is not accessible from previous reference-genome-based studies. For example, we noted that all the *bHLH*s that resulted from WGD/segmental duplication belonged to the core *bHLH*s, whereas all the dispensable *bHLH*s corresponded to small-scale duplications such as tandem, proximal, or dispersed duplications. This observation suggests that genes located in a strictly conserved genetic region are more likely to have an essential function, which makes good biological sense. In addition, we found that dispensable *bHLH*s have experienced clear relaxation of selection pressure compared with core *bHLHs*, an observation consistent with their conservation levels. Similar findings were observed in our previous study (Jia et al., 2023). At the transcript level, we also noticed that all of the universally highly transcribed *bHLH*s were core *bHLH*s, whereas the transcription of dispensable *bHLH*s tended to be weaker and more tissue specific. Nonetheless, we found that over half of the newly predicted bHLHs were actively transcribed.

On the basis of their conservation levels, we speculate that the softcore *bHLH*s may correspond to natural mutations resulting from gene loss events, whereas the shell and cloud bHLHs may have arisen from recent gene duplication and expansion events,

both of which are common phenomena during gene evolution (Albalat and Canestro, 2016). Because softcore *bHLHs show a* high conservation level, their loss in individual barley lines may have significant phenotypic consequences, a possibility that is deserving of special attention in future gene functional studies. By contrast, we found that most of the line-specific *bHLH*s were dispersed gene duplicates. Of the 61 dispensable *bHLH*s identified in this study, line-specific *bHLH*s (24) accounted for approximately one-third, corresponding to an average of ~1.2 specific *bHLH*s per barley genome. These observations suggest that the *bHLH* gene family may be undergoing active duplication in the barley genome, consistent with previous reports that gene duplication may occur as frequently as common genetic variations such as SNPs (Lynch and Conery, 2000).

To date, our understanding of the biological functions of individual *bHLH*s in barley and other plants remains highly fragmentary. Here, we performed an exhaustive literature search on currently characterized bHLHs from different plants (Table 1) and assigned them to specific subfamilies identified in this study. Plant bHLHs have been shown to regulate various biological processes and traits, including organ/cell development, tolerance to various stresses, nutrient absorption, and secondary metabolism. On the basis of their reported biological functions, we attempted to deduce the biological impact of *bHLH* CNVs on barley. For example, SF27 and SF28, which show biased expansion, have been shown to be responsible for increasing abscisic acid (ABA) content in guard cells, mediating salt-stress tolerance, or regulating iron distribution in roots and shoots (Wang et al., 2013a; Song et al., 2022). This suggests that the specific expansion of these dispensable *bHLH*s in barley may be directly related to environmental adaptation. In addition, CNV and gPAV of the softcore bHLH *HvbHLH.SC152*, which also belongs to the SF28 subgroup, may contribute to variation in stress tolerance among the 20 barley lines. We detected 9 shell bHLHs that were not annotated in any barley genome. Five of these shell *bHLH*s (*HvbHLH.SH162-SF03*, *HvbHLH.SH170-SF28*, *HvbHLH.SH177-SF27*, *HvbHLH.SH178-SF01*, and *HvbHLH.SH180-SF28*) were present in more than 10 of the 20 barley genomes. Besides SF27 and SF28, SF01 *bHLH*s have also been reported to have similar functions in stress tolerance and ion absorption and may therefore also have important effects on stress tolerance. By contrast, only 1 SF03 *bHLH* has been characterized; it was shown to function in the formation of an endoplasmic reticulum structure in *Arabidopsis* (Matsushima et al., 2004). The biological function of the highly expanded barley SF03 subfamily remains to be studied to understand the biological impact of its CNV. The other 4 newly predicted shell *bHLHs* (*HvbHLH.SH56*, *HvbHLH.SH159*, *HvbHLH.SH163*, and *HvbHLH.SH179*) were present in 3–9 genomes and belong to SF27, SF13, SF28, and SF23, respectively. Notably, SF23 was identified as a single gene (*HvbHLH.SH179*) in barley and was present in only 6 of the 20 barley genomes. In this case, the selection of a single reference genome would very likely lead to the false conclusion that SF23 is not present in barley, which could be corrected if the pangenome were used as the reference. In *Arabidopsis*, SF23 has been shown to participate in root apical meristem cell divisions and xylem development (Ohashi-Ito et al., 2013, 2014). Because it is a shell gene in barley, this gene may not be essential in barley or may have redundant functions with other gene(s). Interestingly, of the 20 line-specific/cloud *bHLH*s, most of which were newly predicted in this study, 16 were also assigned to SF13, SF27, and SF28, further highlighting the importance of these 3 subfamilies in barley genome evolution and environmental adaptation. The other 4 cloud genes belonged to SF24, SF_like, SF15, and SF02, which have been reported to participate in drought response, salt stress tolerance, and crown root initiation in *Arabidopsis*, rice, and wheat (Kim and Kim, 2006; Todaka et al., 2012; Wang et al., 2023). The barley genomes that contain these cloud genes may be exceptionally resilient to stress conditions, a possibility that warrants attention in future studies. Taken together, our results suggest that dividing genes into different groups on the basis of their conservation levels can provide critical insights into gene evolution, further highlighting the advantages of pangenome-wide gene-family analyses over traditional reference-genome-based studies.

Biased expansion of the *bHLH* gene family in barley

Gene duplication is the major mechanism of gene-family expansion, which plays an important role in plant genome evolution and phenotypic diversification (Flagel and Wendel, 2009). Depending on their genetic mechanisms of origin, gene duplicates can be divided into WGD/segmental, tandem, proximal, and dispersed duplicates (Wang et al., 2012). In this study, we found that all the WGD/segmental duplicated *bHLH*s belonged to the core *bHLH*s, which were strictly conserved in all 20 barley genomes. By contrast, the small-scale *bHLH* duplicates contain core and dispensable *bHLH*s. In particular, all the dispensable *bHLH*s correspond to small-scale duplications. Our recent study suggested that partially retained genes (i.e., dispensable genes) may represent gene duplicates under active selection toward gene fixation in the population (Jia et al., 2022). Therefore, these results suggest that expansion of the *bHLH* gene family in barley is mainly active through small-scale duplications. Future studies will be needed to determine whether dispensable genes in other gene families or species are also more likely to result from small-scale duplications. Several studies have reported that the evolution of the *bHLH* gene family displays a clear species-specific expansion pattern, leading to the identification of species-specific *bHLH* lineages (Miao et al., 2020; Ndayambaza et al., 2021; Zhao et al., 2021). The identification of core and dispensable *bHLH*s in the present study provides a novel perspective on *bHLH* evolution at the population scale. It would be interesting to investigate the genetic mechanisms that underlie the expansion of *bHLH* gene families, which could improve our understanding of *bHLH* gene evolution in plants.

One notable observation in this study was that dispensable *bHLH*s were enriched in specific subfamilies, such as SF13b, SF27b, and SF28b. This finding suggests that the expansion of *bHLH*s in barley may be biased toward the evolution of specific biological functions. On the basis of previously reported *bHLH*s, SF27 and SF28 are generally involved in regulating ABA content in guard cells, improving salt and drought stress tolerance, and regulating iron distribution in roots and shoots (Wang et al., 2013b; Song et al., 2022). These subfamilies include several newly predicted shell bHLHs, and their exceptional expansion in barley may play an important role in its environmental adaptation. Most of these SF13b, SF27b, and SF28b members

were actively transcribed in multiple tissues in our pangenome data, providing further support for their biological importance in barley. A previous study showed that *OsbHLH133* in SF28 can enhance iron concentration in roots but has the opposite effect in shoots and may influence iron usage efficiency under stress conditions such as neutral and alkaline soils (Wang et al., 2013c). It is possible that the specific expansion of SF28 may be related to the adaptation of barley to harsh environmental conditions compared with rice. The function of SF13b remains speculative owing to its unique protein sequence profile. A Gene Ontology prediction and homology search failed to produce any significant hits in public databases (UniProt and InterProScan). Although motif 1 in the bHLH domain is strictly conserved in SF13b, motif 7 in SF13b seems to vary from motif 2 in the bHLH domain. The protein sequence outside the putative bHLH domain in SF13b also varies significantly from that of other bHLHs, suggesting that SF13b may have evolved via gene recombination. Future gene functional studies are needed to investigate the functions of the dispensable SF13b genes.

In conclusion, we took gPAVs into consideration and developed an innovative pangenome-based approach to gene-family characterization. We overcame genome annotation bias and identified 3411 bHLHs in 20 barley pangenome accessions. These bHLHs could be classified into 201 OGGs, more than the number of bHLHs in the reference genome (161). The 201 *bHLH* OGGs were classified into 140 core, 12 softcore, 29 shell, and 20 line-specific/cloud *bHLH*s, revealing the complete profile of *bHLH*s in barley. We identified 3 novel *bHLH* clusters in the specific subfamilies SF13, SF27, and SF28, enriched with dispensable *bHLH*s, implying that the expansion of *bHLHs* in barley may be biased toward specific biological functions. We found that 50% of the *bHLH*s contained at least 1 intact TE within the 2-kb upstream to 2-kb downstream region. *bHLH*s with CNVs tended to have significantly more TEs than those without CNVs, supporting a potential role for TEs in *bHLH* expansion. Core *bHLH*s were more likely to be derived from WGD/segmental duplications, whereas dispensable bHLHs were mainly small-scale duplicates. Analyses of natural selection revealed a clear relaxation of selection pressure on dispensable *bHLH*s. We provided an effective template for the integration of pangenome data with pantranscriptome data to dissect transcriptional patterns across different *bHLH* subfamilies. We conclude that pangenome-wide gene-family analyses can better describe the evolutionary status of genes in a species than traditional reference-genome-based studies.

MATERIALS AND METHODS

Identification of *bHLH* genes in the barley pangenome

The genomic sequences of 20 barley pangenome accessions (Jayakodi et al., 2020) were downloaded from the IPK database (https://barley-pangenome.ipk-gatersleben.de). Comprehensive genome scanning of bHLHs was performed using the BITACORA tool (Vizueta et al., 2020) in the full mode with an E-value of 1e−5 for both BLAST and hmmscan from the HMMER package. The hidden Markov model profile of the bHLH domain (PF00010) was downloaded from http://ftp.ebi.ac.uk/pub/databases/Pfam/releases/Pfam35.0/; it was used together with the genome sequences, gene annotation model files, and annotated bHLH protein sequences in Morex version 2 as input for BITACORA to scan the 20 barley accessions. Well-curated protein sequences of rice bHLHs were retrieved from RGAP (http://rice.plantbiology.msu.edu/) and used for a bHLH domain (PF00010) scan with the hmmscan tool. The identified significant bHLH hits were validated for the presence of a full bHLH domain through a separate hmmscan search and were filtered by bHLH domain $\geq$30 aa.

Phylogenetic tree construction

The conserved bHLH domain sequences were identified using the hmmscan tool in HMMER (http://hmmer.org/). The aa sequences for the bHLH domain were retrieved and aligned using Muscle (8 iterations) (Edgar, 2004). An unrooted maximum-likelihood tree was constructed using IQ-TREE (version 1.6.12) (Nguyen et al., 2015) with the best-fit JTT+G4 substitution model (lowest Bayesian Information Criterion score). Branch support was calculated using 1000 bootstrap replicates and the Shimodaira–Hasegawa-like approximate likelihood ratio test. Tree annotation was performed with FigTree (version 1.4.3, http://tree.bio.ed.ac.uk/software/figtree).

Analysis of gene structures and conserved motifs

The intron–exon structures of the *bHLH*s were retrieved from the gene annotation GFF3 file (Morex version r2). For *bHLH*s absent in Morex, gene annotation files from other barley varieties were used. For newly predicted *bHLH*s, gene structure information obtained with BITACORA was used. The final gene structure information was visualized using TBtools (Chen et al., 2020). Conserved protein motifs (total number set to 10) were identified using MEME (version 5.4.1) with default settings at https://meme-suite.org/meme/tools/meme.

Analysis of gene duplication and synteny

The gene duplication type and syntenic relationships of *bHLH*s were analyzed using MCScanX (Wang et al., 2013c). The protein sequences for each of the 20 barley pangenome accessions were used for all-vs.-all BLASTP analyses (E-value threshold 1e−5) separately. Only the top 5 BLASTP hits were retained following the tool's instructions. The BLASTP output, together with the GFF3 gene-position files (converted into bed format), were used as input for MCScanX. The gene duplication type was inferred using the duplicate_gene_classifier program. The brief procedure is as follows: (1) all genes are initially classified as singletons; (2) genes with BLASTP hits to other genes are re-labeled as dispersed duplicates; (3) for any BLASTP hit, the 2 genes are re-labeled as proximal duplicates if they have a difference in gene rank <20 (configurable); (4) the 2 genes are re-labeled as tandem duplicates if they have a difference in gene rank = 1; (5) anchor genes in collinear blocks are re-labeled as WGD/segmental. Synteny was visualized using the Circos program (Krzywinski et al., 2009).

TE identification

TEs were identified in the pseudomolecule sequences of the 20 barley pangenome accessions using the HiTE program (Hu et al., 2024) with default parameters. Only intact TEs were used

for downstream analyses. TEs were overlapped with *bHLH* genes using the bedtools program (Quinlan and Hall, 2010).

Transcriptome analyses

Raw transcriptome sequencing data for 5 tissues (caryopsis, coleoptile, inflorescence, root, and shoot) of 20 barley genomes were retrieved from a recently published barley pantranscriptome study (Waugh et al., 2024). K-mer-based transcript quantification was performed using the kallisto program (Bray et al., 2016). Mean TPM values and SDs for each gene and tissue across 20 pangenome in different tissues were calculated. Mean TPM values were normalized as log_2(TPM+1). Relative SD was calculated as SD divided by the mean TPM, followed by log_2(std+1) transformation. The expression heatmap was generated using the pheatmap (version 1.0.12) package in R.

Inference of orthologous gene groups

Orthologous *bHLH* gene clusters in the 20 barley pangenome accessions were inferred using the CD-HIT tool (Li and Godzik, 2006) with a cutoff of 95% identity and 90% alignment coverage for longer sequences. The longest protein from each cluster was identified as the representative sequence.

Analysis of natural selection

To calculate pair-wise gene *Ka*, *Ks*, and *Ka/Ks* values, the ParaAT tool (Zhang et al., 2012) was used with default settings. Orthologous *bHLH* gene pairs among the barley pangenome accessions were used as input for ParaAT. Mean *Ka*, *Ks*, *Ka/Ks*, and SD values were calculated using the groupby function in the Pandas package in Python 3.7.

Data plotting and statistical analysis

Bar plots and line plots were generated using Seaborn (version 0.11.2) packages in Python 3.7. Two-way *t* tests were performed using the Scipy (version 1.10.1) Python package. Lines connecting the gene phylogeny and pheatmap cladogram were drawn using the cophyloplot function in the ape (version 5.5) R package in Rstudio (version 1.3.1093).

FUNDING

This work was supported by the Australia Grain Research and Development Corporation (9176507).

ACKNOWLEDGMENTS

We acknowledge the barley genome research community for making the barley pangenome data publicly available. No conflict of interest is declared.

AUTHOR CONTRIBUTIONS

C.L. and Y.J. supervised and conceived the study. C.T. and Y.J. wrote the manuscript. H.H. and Z.Z. edited the manuscript. C.T. was responsible for the literature search and performed the phylogenetic, protein motif, gene structure, and CNV analyses. Y.J., C.T., H.H., and Z.Z. performed the gene scanning, gene duplication, TE, and natural selection analyses. B.C. performed the transcriptome analyses. All authors have read the manuscript.

SUPPLEMENTAL INFORMATION

Supplemental information is available at *Plant Communications Online*.

Received: June 28, 2024
Revised: August 21, 2024
Accepted: November 8, 2024
Published: November 9, 2024

REFERENCES

Albalat, R., and Cañestro, C. (2016). Evolution by gene loss. Nat. Rev. Genet. **17**:379–391.

Bailey, P.C., Martin, C., Toledo-Ortiz, G., Quail, P.H., Huq, E., Heim, M.A., Jakoby, M., Werber, M., and Weisshaar, B. (2003). Update on the basic helix-loop-helix transcription factor gene family in Arabidopsis thaliana. Plant Cell **15**:2497–2502.

Bayer, P.E., Golicz, A.A., Scheben, A., Batley, J., and Edwards, D. (2020). Plant pan-genomes are the new reference. Nat. Plants **6**:914–920.

Bray, N.L., Pimentel, H., Melsted, P., and Pachter, L. (2016). Near-optimal probabilistic RNA-seq quantification. Nat. Biotechnol. **34**:525–527.

Carretero-Paulet, L., Galstyan, A., Roig-Villanova, I., Martínez-García, J.F., Bilbao-Castro, J.R., and Robertson, D.L. (2010). Genome-Wide Classification and Evolutionary Analysis of the bHLH Family of Transcription Factors in Arabidopsis, Poplar, Rice, Moss, and Algae. Plant Physiol. **153**:1398–1412.

Chen, C., Chen, H., Zhang, Y., Thomas, H.R., Frank, M.H., He, Y., and Xia, R. (2020). TBtools: An Integrative Toolkit Developed for Interactive Analyses of Big Biological Data. Mol. Plant **13**:1194–1202.

Della Coletta, R., Qiu, Y., Ou, S., Hufford, M.B., and Hirsch, C.N. (2021). How the pan-genome is changing crop genomics and improvement. Genome Biol. **22**:3.

Edgar, R.C. (2004). MUSCLE: multiple sequence alignment with high accuracy and high throughput. Nucleic Acids Res. **32**:1792–1797.

Fan, Y., Lai, D., Yang, H., Xue, G., He, A., Chen, L., Feng, L., Ruan, J., Xiang, D., Yan, J., et al. (2021). Genome-wide identification and expression analysis of the bHLH transcription factor family and its response to abiotic stress in foxtail millet (Setaria italica L.). BMC Genom. **22**:778.

Feller, A., Machemer, K., Braun, E.L., and Grotewold, E. (2011). Evolutionary and comparative analysis of MYB and bHLH plant transcription factors. Plant J. **66**:94–116.

Flagel, L.E., and Wendel, J.F. (2009). Gene duplication and evolutionary novelty in plants. New Phytol. **183**:557–564.

Gao, L., Gonda, I., Sun, H., Ma, Q., Bao, K., Tieman, D.M., Burzynski-Chang, E.A., Fish, T.L., Stromberg, K.A., Sacks, G.L., et al. (2019). The tomato pan-genome uncovers new genes and a rare allele regulating fruit flavor. Nat. Genet. **51**:1044–1051.

Haas, M., Schreiber, M., and Mascher, M. (2019). Domestication and crop evolution of wheat and barley: Genes, genomics, and future directions. J. Integr. Plant Biol. **61**:204–225.

Hanada, K., Zou, C., Lehti-Shiu, M.D., Shinozaki, K., and Shiu, S.H. (2008). Importance of lineage-specific expansion of plant tandem duplicates in the adaptive response to environmental stimuli. Plant Physiol. **148**:993–1003.

Hao, Y., Zong, X., Ren, P., Qian, Y., and Fu, A. (2021). Basic Helix-Loop-Helix (bHLH) Transcription Factors Regulate a Wide Range of Functions in Arabidopsis. Int. J. Mol. Sci. **22**:7152.

Hu, K., Ni, P., Xu, M., Zou, Y., Chang, J., Gao, X., Li, Y., Ruan, J., Hu, B., and Wang, J. (2024). HiTE: a fast and accurate dynamic boundary adjustment approach for full-length transposable element detection and annotation. Nat. Commun. **15**:5573.

Jayakodi, M., Padmarasu, S., Haberer, G., Bonthala, V.S., Gundlach, H., Monat, C., Lux, T., Kamal, N., Lang, D., Himmelbach, A., et al.

(2020). The barley pan-genome reveals the hidden legacy of mutation breeding. Nature **588**:284–289.

Jia, Y., Xu, M., Hu, H., Chapman, B., Watt, C., B, B., Han, N., Zhu, M., Bian, H., Li, C., et al. (2022). Comparative pangenome-based gene retention analysis in Triticeae reveals factors affecting gene retention following duplication. BMC Biology **21**:25.

Jia, Y., Xu, M., Hu, H., Chapman, B., Watt, C., Buerte, B., Han, N., Zhu, M., Bian, H., Li, C., et al. (2023). Comparative gene retention analysis in barley, wild emmer, and bread wheat pangenome lines reveals factors affecting gene retention following gene duplication. BMC Biol. **21**:25.

Ke, Q., Tao, W., Li, T., Pan, W., Chen, X., Wu, X., Nie, X., and Cui, L. (2020). Genome-wide Identification, Evolution and Expression Analysis of Basic Helix-loop-helix (bHLH) Gene Family in Barley (*Hordeum vulgare L.)*. Curr. Genom. **21**:621–644.

Ke, Y.Z., Wu, Y.W., Zhou, H.J., Chen, P., Wang, M.M., Liu, M.M., Li, P.F., Yang, J., Li, J.N., and Du, H. (2020). Genome-wide survey of the bHLH super gene family in Brassica napus. BMC Plant Biol. **20**:115.

Kim, J., and Kim, H.Y. (2006). Functional analysis of a calcium-binding transcription factor involved in plant salt stress signaling. FEBS Lett. **580**:5251–5256.

Krzywinski, M., Schein, J., Birol, I., Connors, J., Gascoyne, R., Horsman, D., Jones, S.J., and Marra, M.A. (2009). Circos: An information aesthetic for comparative genomics. Genome Res. **19**:1639–1645.

Li, J., Wang, T., Han, J., and Ren, Z. (2020). Genome-wide identification and characterization of cucumber bHLH family genes and the functional characterization of CsbHLH041 in NaCl and ABA tolerance in Arabidopsis and cucumber. BMC Plant Biol. **20**:272.

Li, W., and Godzik, A. (2006). Cd-hit: a fast program for clustering and comparing large sets of protein or nucleotide sequences. Bioinformatics **22**:1658–1659.

Li, X., Duan, X., Jiang, H., Sun, Y., Tang, Y., Yuan, Z., Guo, J., Liang, W., Chen, L., Yin, J., et al. (2006). Genome-wide analysis of basic/helix-loop-helix transcription factor family in rice and Arabidopsis. Plant Physiol. **141**:1167–1184.

Lynch, M., and Conery, J.S. (2000). The evolutionary fate and consequences of duplicate genes. Science **290**:1151–1155.

Ma, H., Wang, M., Zhang, Y.E., and Tan, S. (2023). The power of "controllers": Transposon-mediated duplicated genes evolve towards neofunctionalization. J. Genet. Genom. **50**:462–472.

Marks, R.A., Hotaling, S., Frandsen, P.B., and VanBuren, R. (2021). Representation and participation across 20 years of plant genome sequencing. Nat. Plants **7**:1571–1578.

Maron, L.G., Guimarães, C.T., Kirst, M., Albert, P.S., Birchler, J.A., Bradbury, P.J., Buckler, E.S., Coluccio, A.E., Danilova, T.V., Kudrna, D., et al. (2013). Aluminum tolerance in maize is associated with higher MATE1 gene copy number. Proc. Natl. Acad. Sci. USA **110**:5241–5246.

Matsushima, R., Fukao, Y., Nishimura, M., and Hara-Nishimura, I. (2004). NAI1 gene encodes a basic-helix-loop-helix-type putative transcription factor that regulates the formation of an endoplasmic reticulum-derived structure, the ER body. Plant Cell **16**:1536–1549.

Miao, L., Gao, Y., Zhao, K., Kong, L., Yu, S., Li, R., Liu, K., and Yu, X. (2020). Comparative analysis of basic helix-loop-helix gene family among Brassica oleracea, Brassica rapa, and Brassica napus. BMC Genom. **21**:178.

Ndayambaza, B., Jin, X., Min, X., Lin, X., Yin, X., and Liu, W. (2021). Genome-Wide Identification and Expression Analysis of the Barrel Medic (Medicago truncatula) and Alfalfa (Medicago sativa L.) Basic Helix-Loop-Helix Transcription Factor Family Under Salt and Drought Stresses. J. Plant Growth Regul. **40**:2058–2078.

Nguyen, L.T., Schmidt, H.A., von Haeseler, A., and Minh, B.Q. (2015). IQ-TREE: A Fast and Effective Stochastic Algorithm for Estimating Maximum-Likelihood Phylogenies. Mol. Biol. Evol. **32**:268–274.

Niu, X., Guan, Y., Chen, S., and Li, H. (2017). Genome-wide analysis of basic helix-loop-helix (bHLH) transcription factors in Brachypodium distachyon. BMC Genom. **18**:619.

Ohashi-Ito, K., Matsukawa, M., and Fukuda, H. (2013). An Atypical bHLH Transcription Factor Regulates Early Xylem Development Downstream of Auxin. Plant Cell Physiol. **54**:398–405.

Ohashi-Ito, K., Saegusa, M., Iwamoto, K., Oda, Y., Katayama, H., Kojima, M., Sakakibara, H., and Fukuda, H. (2014). A bHLH Complex Activates Vascular Cell Division via Cytokinin Action in Root Apical Meristem. Curr. Biol. **24**:2053–2058.

Quinlan, A.R., and Hall, I.M. (2010). BEDTools: a flexible suite of utilities for comparing genomic features. Bioinformatics **26**:841–842.

Robertson, F.M., Gundappa, M.K., Grammes, F., Hvidsten, T.R., Redmond, A.K., Lien, S., Martin, S.A.M., Holland, P.W.H., Sandve, S.R., and Macqueen, D.J. (2017). Lineage-specific rediploidization is a mechanism to explain time-lags between genome duplication and evolutionary diversification. Genome Biol. **18**:111.

Sarkar, T., Thankappan, R., Mishra, G.P., and Nawade, B.D. (2019). Advances in the development and use of *DREB* for improved abiotic stress tolerance in transgenic crop plants. Physiol. Mol. Biol. Plants **25**:1323–1334.

Schiessl, S.V., Katche, E., Ihien, E., Chawla, H.S., and Mason, A.S. (2019). The role of genomic structural variation in the genetic improvement of polyploid crops. Crop J. **7**:127–140.

Shen, T., Wen, X., Wen, Z., Qiu, Z., Hou, Q., Li, Z., Mei, L., Yu, H., and Qiao, G. (2021). Genome-wide identification and expression analysis of bHLH transcription factor family in response to cold stress in sweet cherry (Prunus avium L.). Sci. Hortic. **279**:109905.

Song, Y., Li, S., Sui, Y., Zheng, H., Han, G., Sun, X., Yang, W., Wang, H., Zhuang, K., Kong, F., et al. (2022). SbbHLH85, a bHLH member, modulates resilience to salt stress by regulating root hair growth in sorghum. Theor. Appl. Genet. **135**:201–216.

Sun, H., Fan, H.J., and Ling, H.Q. (2015). Genome-wide identification and characterization of the bHLH gene family in tomato. BMC Genom. **16**:9.

Sun, P.W., Gao, Z.H., Lv, F.F., Yu, C.C., Jin, Y., Xu, Y.H., and Wei, J.H. (2022a). Genome-wide analysis of basic helix-loop-helix (bHLH) transcription factors in Aquilaria sinensis. Sci. Rep. **12**:7194.

Sun, X., Wang, Y., and Sui, N. (2018). Transcriptional regulation of bHLH during plant response to stress. Biochem. Biophys. Res. Commun. **503**:397–401.

Sun, Y. (2022). Pangenome analysis reveals the abundant gene presence/absence variations (PAVs) among different varieties of melon and their influence on traits. Front. Plant Sci. **13**:835496.

Sun, Y., Zhang, P.T., Kou, D.R., Han, Y.C., Fang, J.C., Ni, J.P., Jiang, B., Wang, X., Zhang, Y.J., Wang, W., et al. (2022b). Terpene Synthases in Rice Pan-Genome and Their Responses to *Chilo suppressalis* Larvae Infesting. Front. Plant Sci. **13**:905982.

Swanson-Wagner, R.A., Eichten, S.R., Kumari, S., Tiffin, P., Stein, J.C., Ware, D., and Springer, N.M. (2010). Pervasive gene content variation and copy number variation in maize and its undomesticated progenitor. Genome Res. **20**:1689–1699.

Tettelin, H., Masignani, V., Cieslewicz, M.J., Donati, C., Medini, D., Ward, N.L., Angiuoli, S.V., Crabtree, J., Jones, A.L., Durkin, A.S., et al. (2005). Genome analysis of multiple pathogenic isolates of Streptococcus agalactiae: Implications for the microbial "pan-genome. Proc. Natl. Acad. Sci. USA **102**:13950–13955.

Todaka, D., Nakashima, K., Maruyama, K., Kidokoro, S., Osakabe, Y., Ito, Y., Matsukura, S., Fujita, Y., Yoshiwara, K., Ohme-Takagi, M., et al. (2012). Rice phytochrome-interacting factor-like protein OsPIL1 functions as a key regulator of internode elongation and induces a morphological response to drought stress. Proc. Natl. Acad. Sci. USA **109**:15947–15952.

Toledo-Ortiz, G., Huq, E., and Quail, P.H. (2003). The Arabidopsis basic/helix-loop-helix transcription factor family. Plant Cell **15**:1749–1770.

Van de Weyer, A.L., Monteiro, F., Furzer, O.J., Nishimura, M.T., Cevik, V., Witek, K., Jones, J.D.G., Dangl, J.L., Weigel, D., and Bemm, F. (2019). A Species-Wide Inventory of NLR Genes and Alleles in Arabidopsis thaliana. Cell **178**:1260–1272.e14.

Vernikos, G., Medini, D., Riley, D.R., and Tettelin, H. (2015). Ten years of pan-genome analyses. Curr. Opin. Microbiol. **23**:148–154.

Vizueta, J., Sánchez-Gracia, A., and Rozas, J. (2020). bitacora: A comprehensive tool for the identification and annotation of gene families in genome assemblies. Mol. Ecol. Resour. **20**:1445–1452.

Wang, J., Li, C., Mao, X., Wang, J., Li, L., Li, J., Fan, Z., Zhu, Z., He, L., and Jing, R. (2023). The wheat basic helix-loop-helix gene TabHLH123 positively modulates the formation of crown roots and is associated with plant height and 1000-grain weight under various conditions. J. Exp. Bot. **74**:2542–2555.

Wang, L., Ying, Y., Narsai, R., Ye, L., Zheng, L., Tian, J., Whelan, J., and Shou, H. (2013a). Identification of OsbHLH133 as a regulator of iron distribution between roots and shoots in. Plant Cell Environ. **36**:224–236.

Wang, L., Ying, Y., Narsai, R., Ye, L., Zheng, L., Tian, J., Whelan, J., and Shou, H. (2013b). Identification of OsbHLH133 as a regulator of iron distribution between roots and shoots in Oryza sativa. Plant Cell Environ. **36**:224–236.

Wang, P., Su, L., Gao, H., Jiang, X., Wu, X., Li, Y., Zhang, Q., Wang, Y., and Ren, F. (2018a). Genome-Wide Characterization of bHLH Genes in Grape and Analysis of their Potential Relevance to Abiotic Stress Tolerance and Secondary Metabolite Biosynthesis. Front. Plant Sci. **9**:64.

Wang, R., Zhao, P., Kong, N., Lu, R., Pei, Y., Huang, C., Ma, H., and Chen, Q. (2018b). Genome-Wide Identification and Characterization of the Potato bHLH Transcription Factor Family. Genes **9**:54.

Wang, Y., Li, J., and Paterson, A.H. (2013c). MCScanX-transposed: detecting transposed gene duplications based on multiple colinearity scans. Bioinformatics **29**:1458–1460.

Wang, Y., Tang, H., DeBarry, J.D., Tan, X., Li, J., Wang, X., Lee, T.H., Jin, H., Marler, B., Guo, H., et al. (2012). MCScanX: a toolkit for detection and evolutionary analysis of gene synteny and collinearity. Nucleic Acids Res. **40**:e49.

Wang, Z., Jia, C., Wang, J.Y., Miao, H.X., Liu, J.H., Chen, C., Yang, H.X., Xu, B., and Jin, Z. (2020). Genome-Wide Analysis of Basic Helix-Loop-Helix Transcription Factors to Elucidate Candidate Genes Related to Fruit Ripening and Stress in Banana (*Musa acuminata* L. AAA Group, cv. Cavendish). Front. Plant Sci. **11**:650.

Waugh, R., Guo, W., Schreiber, M., Marosi, V., Bagnaresi, P., Chalmers, K., Chapman, B., Dang, V., Dockter, C., Fiebig, A., et al. (2024). A barley pan-transcriptome reveals layers of genotype-dependent transcriptional complexity. Preprint at Researchsquare., 07 January 2024, PREPRINT (Version 1) available at Research Square https://doi.org/10.21203/rs.3.rs-3787876/v1.

Zhang, L.Y., Bai, M.Y., Wu, J., Zhu, J.Y., Wang, H., Zhang, Z., Wang, W., Sun, Y., Zhao, J., Sun, X., et al. (2009). Antagonistic HLH/bHLH Transcription Factors Mediate Brassinosteroid Regulation of Cell Elongation and Plant Development in Rice and Arabidopsis. Plant Cell **21**:3767–3780.

Zhang, T., Lv, W., Zhang, H., Ma, L., Li, P., Ge, L., and Li, G. (2018). Genome-wide analysis of the basic Helix-Loop-Helix (bHLH) transcription factor family in maize. BMC Plant Biol. **18**:235.

Zhang, Z., Xiao, J., Wu, J., Zhang, H., Liu, G., Wang, X., and Dai, L. (2012). ParaAT: A parallel tool for constructing multiple protein-coding DNA alignments. Biochem. Biophys. Res. Commun. **419**:779–781.

Zhang, Z., Chen, J., Liang, C., Liu, F., Hou, X., and Zou, X. (2020). Genome-Wide Identification and Characterization of the bHLH Transcription Factor Family in Pepper (Capsicum annuumL.). Front. Genet. **11**.

Zhao, W., Liu, Y., Li, L., Meng, H., Yang, Y., Dong, Z., Wang, L., and Wu, G. (2021). Genome-Wide Identification and Characterization of bHLH Transcription Factors Related to Anthocyanin Biosynthesis in Red Walnut (*Juglans regia* L.). Front. Genet. **12**:632509.

Zheng, K.J., Wang, Y.T., Zhang, N., Jia, Q.M., Wang, X.T., Hou, C.J., Chen, J.G., and Wang, S.C. (2017). Involvement of PACLOBUTRAZOL RESISTANCE6/KIDARI, an Atypical bHLH Transcription Factor, in Auxin Responses in Arabidopsis. Front. Plant Sci. **8**:1813.

Zhou, X., Liao, Y., Kim, S.U., Chen, Z., Nie, G., Cheng, S., Ye, J., and Xu, F. (2020). Genome-wide identification and characterization of bHLH family genes from Ginkgo biloba. Sci. Rep. **10**:13723.

Plant Communications, Volume *6*

Supplemental information

Pangenome and pantranscriptome as the new reference for gene-family characterization: A case study of basic helix-loop-helix (*bHLH*) genes in barley

Cen Tong, Yong Jia, Haifei Hu, Zhanghui Zeng, Brett Chapman, and Chengdao Li

Supplementary Figures

Supplementary Figure S1. Displays the protein motifs and gene structures of *bHLHs* in barley. **A**. Displays the top 10 conserved protein motifs. Scale bar indicates amino acid (aa) sequence length. **B**. Displays the gene structure (exon and intron). Scale bar indicates gene sequence length in thousand base pair (kb). Target *bHLHs* were sorted by a maximum likelihood phylogenetic tree (left), with different subfamilies highlighted in same colors as in Figure 2.

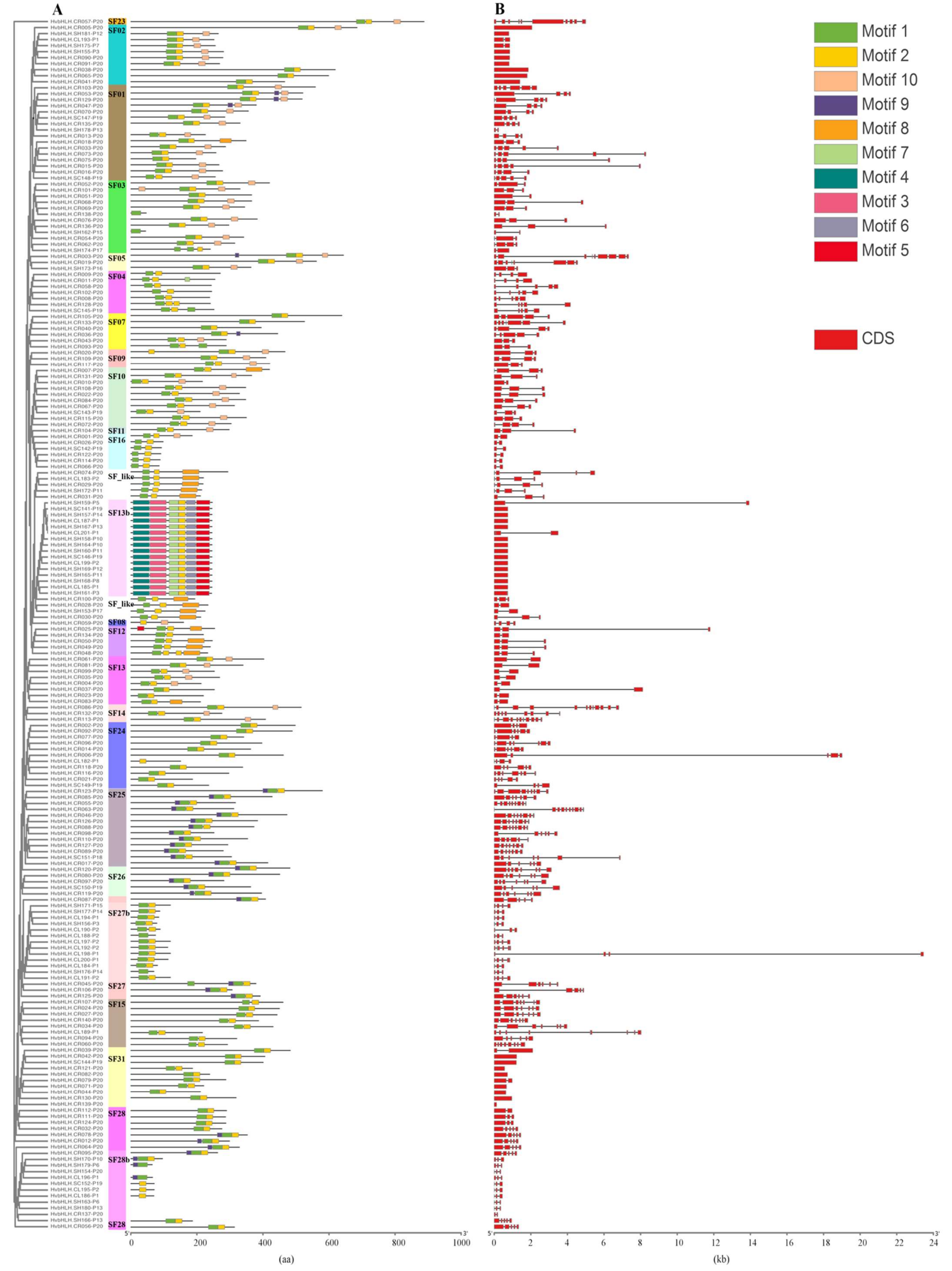

Supplementary Figure S2. Displays the chromosome location, synteny relationship, and gene duplication type of bHLHs in Morex. bHLHs located in conserved collinear regions were linked by red line. Based on gene duplication mechanisms, bHLHs were classified as whole-genome/segmental duplication (purple), tandem duplication (green), proximal duplication (pink), and dispersed duplication (blue). Newly predicted bHLH gene IDs in this study were highlighted in red.

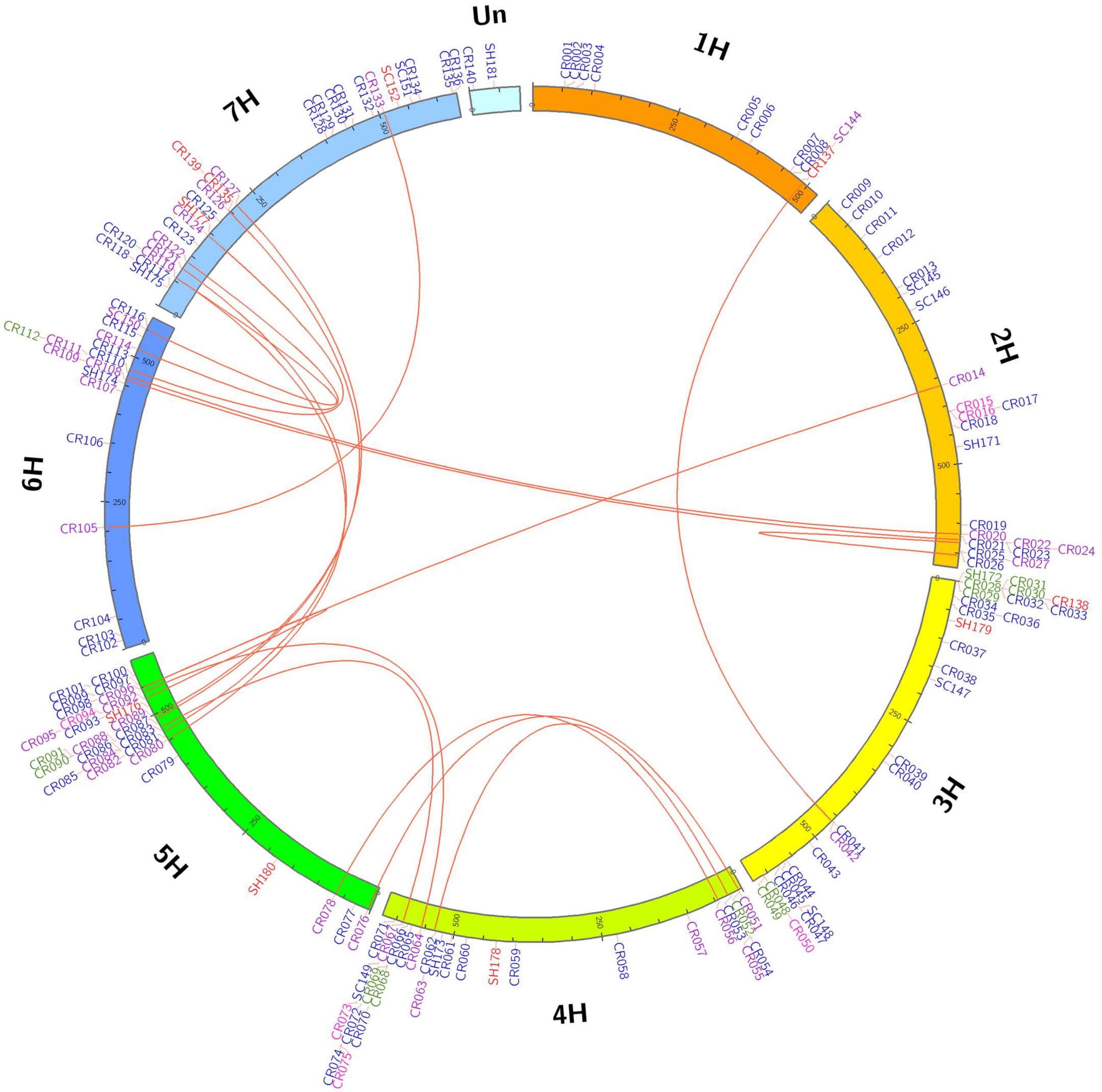

Supplementary Figure S3. Displays the distribution of *Ka*, *Ks*, and *Ka*/*Ks* for each *bHLH* OG (sorted by evolutionary phylogeny). See supplementary file S13 for detailed calculation.

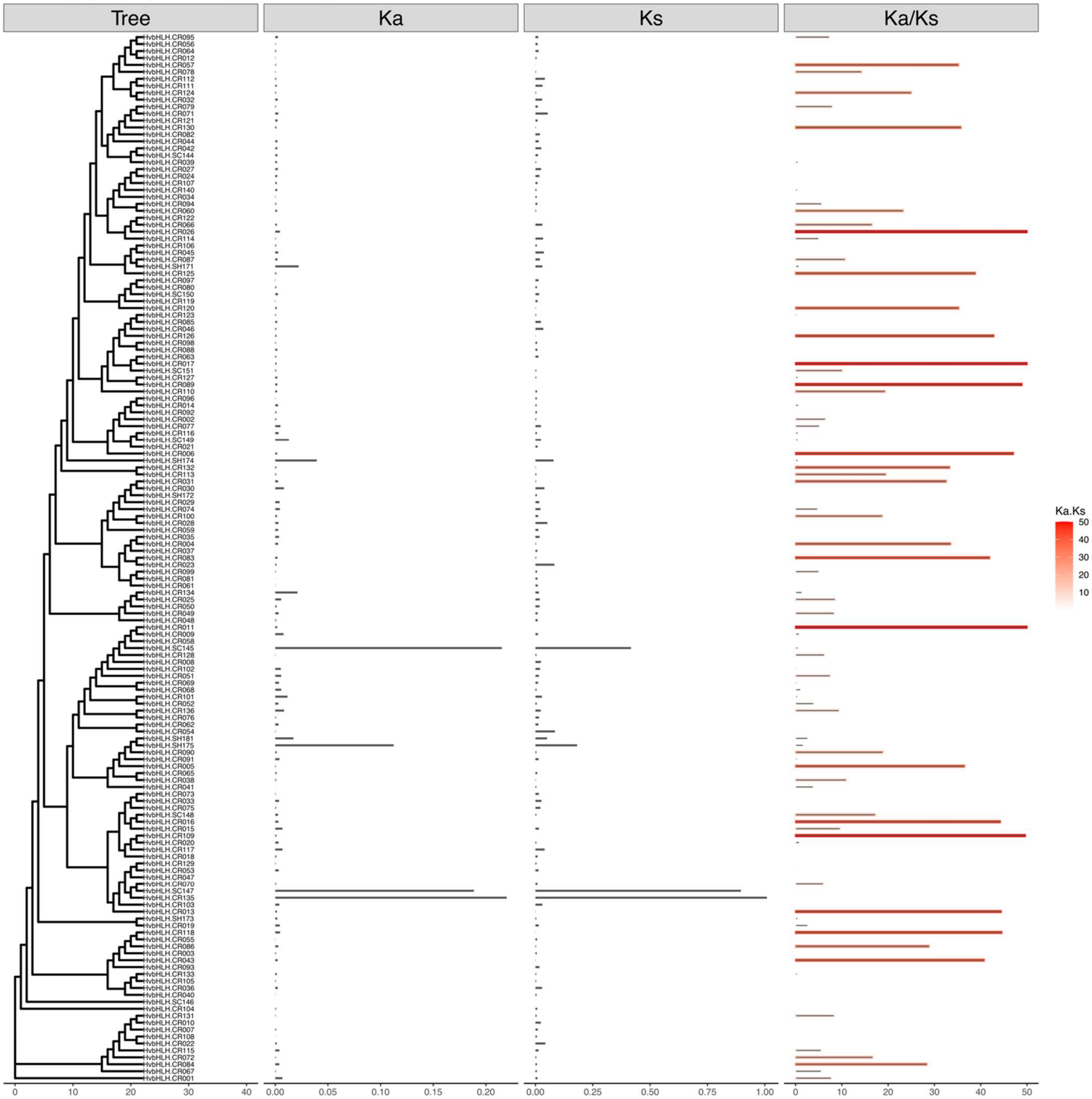

Supplement: Document S2. Article plus supplemental information [file mmc3.pdf]
